# Programmable microwave cluster states via Josephson metamaterials

**DOI:** 10.1038/s41467-026-74377-2

**Published:** 2026-06-18

**Authors:** A. Alocco, A. Celotto, E. Palumbo, B. Galvano, P. Livreri, L. Fasolo, L. Callegaro, E. Enrico

**Affiliations:** 1 Department of Applied Science and Technology, Politecnico di Torino, Turin, Italy; 2 Quantum Metrology and Nanotechnology Division, Istituto Nazionale di Ricerca Metrologica, Turin, Italy; 3 Department of Engineering, Università degli Studi di Palermo, Palermo, Italy

**Keywords:** Single photons and quantum effects, Quantum optics, Superconducting properties and materials

## Abstract

Cluster states are a fundamental resource for continuous-variable quantum computing, enabling measurement-based protocols that can scale beyond the limitations of qubit-based architectures. Here, we demonstrate on-demand generation of multimode entangled microwave cluster states using a programmable Josephson Traveling-Wave Parametric Amplifier (JTWPA) operated in the three-wave mixing regime. By injecting a tailored, non-equidistant set of pump tones via an arbitrary waveform generator, we engineer frequency-specific nonlinear couplings between multiple frequency modes. The entanglement structure is verified via frequency-resolved heterodyne detection of quadrature nullifiers, confirming the target graph topology of the cluster state. Our approach allows reconfigurability through the pumps spectrum and supports scalability by leveraging the wide bandwidth and spatial homogeneity of the JTWPA. This platform opens new avenues for scalable measurement-based quantum information processing in the microwave domain, compatible with superconducting circuit architectures.

## Abstract (web summary)

Microwave cluster states are a key resource for continuous variable quantum computing, but existing platforms offer limited reconfigurability and scalability. Here, the authors report on demand generation of reconfigurable and scalable multimode microwave cluster states using a programmable Josephson traveling wave parametric amplifier, where tailored pump spectra define the graph connectivity.

## Introduction

Quantum information processing with continuous variables (CV) provides a powerful framework for realizing scalable quantum computation, built upon Gaussian states, deterministic operations, and high-efficiency measurements^1,2^. A central resource in this paradigm is the CV cluster state, a highly entangled multipartite state that underpins measurement-based quantum computing (MBQC)^3–5^. In the optical domain, the generation of large-scale cluster states has been successfully demonstrated using time-multiplexed or frequency-encoded modes^5–8^. These implementations typically rely on dielectric nonlinearities and optical interferometry^9^. However, transitioning cluster state generation to the microwave regime holds great promise, particularly in the context of superconducting circuits, both in discrete and CV architectures^10–12^. The microwave platform offers compatibility with circuit quantum electrodynamics (cQED), coherent interaction mechanisms, and the potential for fully integrated and reconfigurable quantum processors^13,14^. Continuous-variable entanglement of microwave modes has been demonstrated with Josephson-junction-based devices^15–17^, including the realization of two-mode squeezing and multipartite configurations^12,18–20^. Nevertheless, these approaches typically involve discrete resonators or fixed topologies, which limit the scalability and flexibility needed for large-scale MBQC. In this work, we present a new platform based on a Josephson Traveling-Wave Parametric Amplifier (JTWPA)^21^ operating in the three-wave mixing regime, which enables the deterministic generation of reconfigurable and scalable cluster states across multiple microwave modes. By driving the JTWPA with a custom-designed set of non-equidistant pump tones, we selectively activate mode couplings corresponding to the desired graph connectivity. The use of broadband traveling-wave interaction allows simultaneous generation of multiple entangled pairs, forming extended cluster graphs. We characterize the resulting quantum correlations by heterodyne detection of frequency-resolved nullifier observables, which serve as witnesses for the underlying entanglement structure^22,23^. Our results demonstrate a flexible and scalable platform for continuous-variable measurement-based quantum computation based on superconducting circuit technologies.

## Results

### Programmable generation of cluster states

**Fig. 1:**
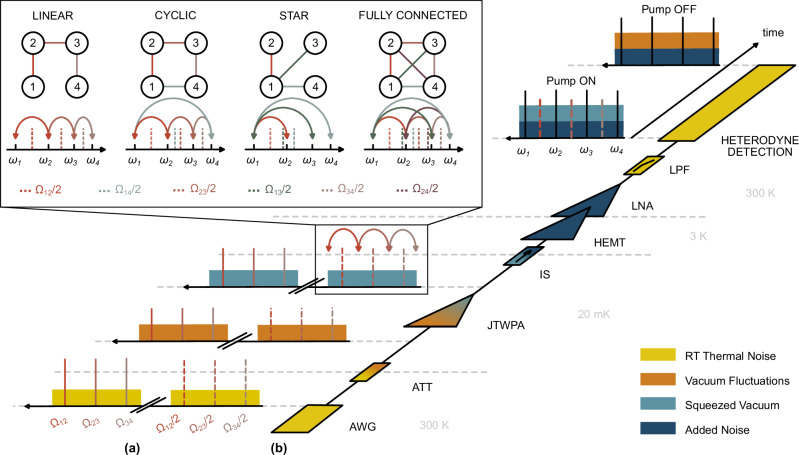
Programmable generation of microwave cluster states using a Josephson Traveling-Wave Parametric Amplifier (JTWPA). **a** Frequency-domain continuous-variable (CV) cluster-state protocol. Multiple non-equidistant pump tones at frequencies Ω_*jl*_ drive the JTWPA in the three-wave mixing regime, implementing two-mode squeezing interactions between vacuum modes at frequencies *ω*_*j*_ and *ω*_*l*_. The interaction transforms input vacuum fluctuations into two-mode squeezed vacuum states, while thermal noise from room-temperature (RT) components is suppressed by cryogenic attenuation. The detection scheme employs a pump ON/OFF reference protocol to subtract the added noise of the readout chain. **b** Block diagram of the experimental setup, including an arbitrary waveform generator (AWG) for pump synthesis, cryogenic attenuators (ATT) to reduce RT thermal noise, the JTWPA, an isolator to prevent back-action, a cryogenic HEMT amplifier followed by a LNA, a low-pass filter (LPF) to suppress pump leakage, and a multiplexed heterodyne detection stage. Inset: example cluster topologies and corresponding pump configurations. Each edge between modes (*j*, *l*) is implemented by applying a pump tone at frequency Ω_*jl*_ = *ω*_*j*_ + *ω*_*l*_.

Continuous-variable (CV) cluster states are defined by a graph structure where each node represents a bosonic mode and edges correspond to entangling interactions^3,4^. In the frequency domain, this structure can be implemented by coupling different frequency modes via nonlinear mixing processes^24,25^. We implement this coupling using a JTWPA operating in the three-wave mixing regime. The effective interaction is described by the Hamiltonian

1 $${\widehat{H}}_{{{\rm{eff}}}}={\sum }_{k=1}^{n}{\sum }_{j\le l}\left({r}_{jl}^{(k)}{\widehat{a}}_{j}^{{\dagger} }{\widehat{a}}_{l}^{{\dagger} }+{r}_{jl}^{(k)*}{\widehat{a}}_{j}{\widehat{a}}_{l}\right),$$

where ${\widehat{a}}_{j}$ and ${\widehat{a}}_{l}$ are the annihilation operators for modes *j* and *l*, with respective frequencies *ω*_*j*_ and *ω*_*l*_. The parameter ${r}_{jl}^{(k)}$ denotes the squeezing amplitude associated with the *k*-th pump tone of frequency ${\Omega }_{jl}^{(k)}$, which satisfies the energy conservation condition ${\omega }_{j}+{\omega }_{l}={\Omega }_{jl}^{(k)}$. The total number of pump tones, *n*, is determined by the chosen coupling topology (see inset in Fig. 1). To enable flexible control over the interaction graph, we synthesize a programmable set of pump tones using an arbitrary waveform generator (AWG). These pumps span the JTWPA’s operational bandwidth and are non-equidistant to match desired pairwise resonances. This method enables arbitrary graph structures among a selected set of frequency modes, akin to an optical frequency comb^5–7^. In this work, we focus on four-mode (*N* = 4) cluster states, exploring several topologies including linear, cyclic, star, and fully connected graphs. Cluster state generation is verified using multiplexed heterodyne detection and nullifier measurements^8,12,23^, allowing reconstruction of the graph structure from measured correlations (see Fig. 1). Further details on the Hamiltonian model and pump-tone selection strategy can be found in Supplementary Notes 2 and 3.

### Cluster state verification

**Fig. 2:**
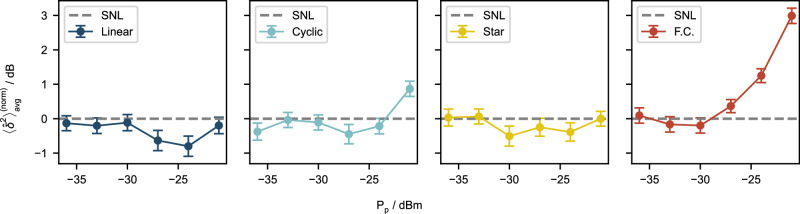
Cluster quality metrics for different topologies and pump powers. Dependence of the average nullifier variance, normalized to the shot noise level (SNL), ${\langle {\widehat{\delta }}^{2}\rangle }_{{{\rm{avg}}}}^{(\,{{\rm{norm}}})}$, on the power *P*_p_ of a single pump tone (identical for all pairs), measured at the room-temperature reference plane, for different cluster topologies. The error bars show the estimated standard deviation.

To verify the presence of multipartite entanglement and validate the underlying graph structure of the generated cluster state, we employ nullifier-based measurements^22,23^. In the continuous-variable model, an ideal cluster state associated with an adjacency matrix **A** satisfies a set of operator equations called nullifiers:

2 $${\widehat{\delta }}_{j}={\widehat{p}}_{j}-{\sum }_{l}{A}_{jl}{\widehat{x}}_{l}\to 0,$$

where ${\widehat{x}}_{l}$ and ${\widehat{p}}_{j}$ are the amplitude and phase quadratures, respectively, of modes *l* and *j*, expressed in the photon basis, and *A*_*jl*_ is the real-valued weight of the connection between modes *j* and *l*. In practice, finite squeezing leads to non-zero nullifier variances. A state is considered a valid cluster state if all nullifier variances are suppressed below the shot noise level (SNL)^26,27^. To experimentally verify this condition, we perform heterodyne detection on each frequency mode *ω*_*j*_. The measurement yields the in-phase (I) and quadrature (Q) voltage components of the microwave field, from which we construct the covariance matrix *Γ* at the JTWPA output, defined on the quadrature vector $R={({x}_{1},{p}_{1},\ldots,{x}_{N},{p}_{N})}^{T}$. Building on this, we compute the nullifier variances using Equation (2). Our results, reported in Fig. 2, show that nullifier variances fall below the SNL across a wide range of pump powers for four different graph topologies, thereby verifying the successful realization of the cluster states. The full derivation of the nullifiers, the construction of the adjacency matrix, and numerical reconstruction techniques are provided in the Supplementary Note 3.

## Discussion

**Fig. 3:**
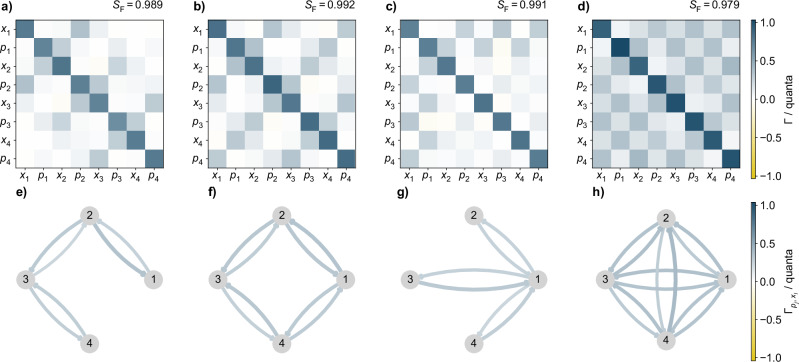
Quadrature covariance matrices and inferred graph connectivity. **a**–**d** Reconstructed covariance matrix Γ of the quadrature operators at *P*_p_ = −24 dBm, extracted from heterodyne detection of four frequency modes clustered in linear (**a**, **e**), cyclic (**b**, **f**), star (**c**, **g**), and fully-connected (**d**, **h**) configurations. **e**–**h** Evaluation of the graph connectivity inferred from the elements of the covariance matrix, with the color of the arrow pointing from the *j*-th to the *l*-th node mapping the value of the ${\Gamma }_{{p}_{j},{x}_{l}}$ term.

The programmability of our approach consists in its ability to reconfigure the cluster-state graph in real time simply by changing the pump spectrum. This capability enables rapid reconfiguration of the interaction graph, which is critical for implementing measurement-based quantum algorithms and quantum error correction in the CV regime^28–30^. The programmability of the cluster states is further confirmed by the corresponding network graphs, which match the programmed topology (see Fig. 3). The reconstructed graphs covariance matrices describing the cluster states are quantitatively compared with theoretical predictions using the normalized Frobenius similarity (*S*_F_) (see Supplementary Equation (S47)), with the worst-case deviation below 3%, demonstrating excellent agreement with the target graph. Combined with high detection efficiency and verified suppression of the nullifier variances across multiple frequency modes, this establishes a practical route toward real-time continuous-variable quantum processing using integrated superconducting hardware.

By encoding quantum modes in the frequency domain and engineering entanglement via pump tone synthesis, we eliminate the need for bulky optical elements or time-multiplexed delay lines traditionally used in CV platforms^6,7,31^.

Compared to conventional cavity-based Josephson parametric amplifiers, our approach enables entanglement over several GHz bandwidth. The programmable nature of the AWG-based pumps allows real-time reconfiguration of the graph structure, making the system well-suited for dynamic CV quantum protocols^31^. In our experiment, we observed nullifier squeezing of 0.8 dB, 0.4 dB, 0.4 dB, and 0.2 dB for the linear, cyclic, star, and fully connected clusters, respectively, with a reduction in squeezing for topologies requiring more pump tones, consistent with previous results in the microwave regime^12,32^. These values fall within the range reported in optical continuous-variable studies for clusters of comparable size: OPO-based implementations reach up to ~3 dB of squeezing for linear, cyclic, and star configurations^23^, while integrated microcomb chips achieve around ~2 dB^8^. Although our microwave platform exhibits lower squeezing, its in-situ programmability and compatibility with superconducting hardware provide a complementary approach toward scalable measurement-based continuous-variable quantum computation.

The achieved nullifier squeezing is sufficient to implement elementary continuous-variable protocols such as entanglement swapping and quantum teleportation in the microwave domain^33^, provided that appropriate Bell-type measurements are performed. By contrast, universal and fault-tolerant CV quantum computation requires substantially larger cluster states (with a number of modes that scales with the circuit width and depth) and higher squeezing (on the order of 15 dB), together with a non-Gaussian resource^28,34–36^. These requirements highlight that significant advances in cluster size and achievable squeezing will be necessary to reach this regime in the microwave domain. Current limitations arise from both the device under test and the measurement chain. At the device level, they include limited squeezing generation^37^, potentially worsened by residual higher order harmonics products that contribute to losses in the JTWPA^32^. A potential improvement is the use of a left-handed JTWPA, which is expected to suppress unwanted mixing processes and achieve higher squeezing levels^38^^39^. Experimental limitations arise from the use of a 3K HEMT as the first amplification stage and from room-temperature pump-tone filtering. These issues could be mitigated by implementing a quantum-limited amplifier as the first stage and by introducing cryogenic pump filtering to improve pump isolation and measurement stability. A 3K HEMT typically adds on the order of 20 noise quanta, whereas quantum-limited amplifiers add a minimum of 1/2 quantum, which would reduce the required acquisition time and mitigate the impact of system drifts^40^. Looking ahead, our architecture is naturally extensible to higher-order graphs and frequency-mode multiplexing with thousands of channels, compatible with ongoing developments in cryogenic AWGs and integrated superconducting photonics^41,42^. The ability to perform adaptive measurements and conditional feedforward using FPGA-based control further opens the path toward full-scale measurement-based quantum computation. Our work thus establishes a step toward linking optical CV platforms and microwave quantum hardware, offering a flexible and hardware-efficient approach for continuous-variable quantum information processing.

## Methods

### Experimental setup

The experimental platform is built around a pre-commercial Al-TWPA-C from Arctic Instruments, consisting of a periodic array of Josephson junction-based meta-atoms embedded in a thin-film waveguide structure^32^. The JTWPA supports broadband non-degenerate three-wave mixing when biased with an external DC flux and driven by a programmable set of pump tones. Pump tones are synthesized using a high-speed AWG and digitally I/Q-modulated onto a microwave carrier before attenuation and injection into the JTWPA input port (see Fig. 1). The broadband vacuum fluctuations act as the quantum seed for spontaneous two-mode squeezing via parametric interactions. The output of the JTWPA is passed through an isolator, low-noise amplifiers, and a room-temperature down-conversion chain before being digitized by a fast analog-to-digital converter. The acquired signals are processed using digital signal processing (DSP) to perform mode demultiplexing and quadrature extraction^18,43^. The experiment is housed in a cryogen-free dilution refrigerator, which operates below 100 mK to minimize thermal noise. Signal integrity is preserved using superconducting coaxial cables, attenuators, and infrared filters at proper temperature stages. Phase coherence between pump, seed, and LO paths is actively stabilized using a reference tone distributed throughout the setup. A comprehensive description of the cryogenic setup, signal routing, and DSP implementation is included in the Supplementary Note 1.

### Covariance matrix reconstruction

**Fig. 4:**
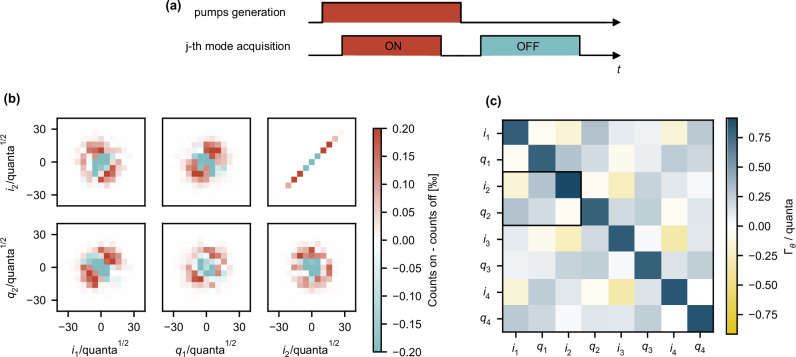
Gaussian cluster state covariance matrix reconstruction. **a** Scheme of the pumps interleaved generation via an ON-OFF protocol, which guarantees referenced-state multiplexed heterodyne detection of modes. **b** Histograms reporting the counts of quadratures measured in the ON condition, subtracted using the reference state acquired during the OFF state for modes 1 and 2. Correlations are reported in the pairs (*i*_2_, *q*_1_) and (*q*_2_, *i*_1_), while anti-correlations are in (*i*_2_, *i*_1_). **c** Covariance matrix in the yet unknown *θ*-rotated basis Γ_*θ*_ reconstructed from multiplexed heterodyne measurements on 4 modes cyclically clustered via 4 pumps at *P*_p_ = −24 dBm. The black box indicates the elements of the covariance matrix corresponding to the data reported in (**b**).

We perform heterodyne detection on each frequency mode, properly tuning a set of Local Oscillators (LO) and exploiting a digital down-conversion scheme to extract the correlations between different quadratures over time windows synchronized with the pump tone cycle. Each acquisition consists of two consecutive stages: one with the pump tones activated (ON) and one with the pumps turned off (OFF), under otherwise identical experimental conditions (see Fig. 4a). By repeating this procedure 5 × 10^6^ times, we reconstruct two covariance matrices, ${{{\mathbf{\Gamma }}}}_{{{\rm{ON}}}}^{\exp }$ and ${{{\mathbf{\Gamma }}}}_{{{\rm{OFF}}}}^{\exp }$, computed from the scaled quadrature vector ${{\boldsymbol{M}}}={({i}_{1},{q}_{1},\ldots,{i}_{N},{q}_{N})}^{T}$, where *i*_*j*_ and *q*_*j*_ are obtained by calibrating the raw in-phase and quadrature voltage signals *I*_*j*_ and *Q*_*j*_ from heterodyne detection into the photon basis. The covariance matrix at the JTWPA output is then obtained via a reference state subtraction method (see Fig. 4b, c)^15,44^:

3 $${{{\mathbf{\Gamma }}}}_{\theta }=\left({{{\mathbf{\Gamma }}}}_{{{\rm{ON}}}}^{\exp }-{{{\mathbf{\Gamma }}}}_{{{\rm{OFF}}}}^{\exp }\right)+{{{\boldsymbol{\sigma }}}}^{{{\bf{in}}}}.$$

Here, ***σ***^**in**^ is the covariance matrix of the thermal state at cryogenic temperature. From the reconstructed covariance matrix **Γ**_*θ*_, we recover the cluster state covariance matrix Γ in the unrotated quadrature basis ${{\boldsymbol{R}}}={({x}_{1},{p}_{1},\ldots,{x}_{N},{p}_{N})}^{T}$ via numerical optimization of local phase rotations (see Fig. 3b–f), maximizing the off-diagonal *p* − *x* correlations. Details on the derivation of **Γ** are provided in the Supplementary Note 4.

## Supplementary information

Supplementary Information

Transparent Peer Review file

## Source data

Source Data

## Data Availability

The experimental data generated in this study are provided in the Source Data file. Simulated data were generated based on the theoretical model described in the Supplementary Note 3; the model equations and parameters are provided to allow independent reproduction of the simulations. Data underlying Supplementary Figures are available from the corresponding author upon request. Source data are provided with this paper.
