# Supplementary material for: Programmable microwave cluster states via Josephson metamaterials
Source: Nat Commun. 2026 Jun 18;17:7700. doi: 10.1038/s41467-026-74377-2 (PMC13434799; doi:10.1038/s41467-026-74377-2)
Supplement: Supplementary file 1 [file 41467_2026_74377_MOESM1_ESM.pdf]

Programmable Microwave Cluster States via Josephson Metamaterials

Contents

1 Detailed Experimental Scheme **6**

2 JTWPA Details and Pumps and Modes Schemes Selection **7**
- 2.1 JTWPA details 7
- 2.2 JTWPA Gain Flatness 7
- 2.3 JTWPA two-mode squeezing generation 8
- 2.4 Spectral Engineering for Cluster State Generation 9
- 2.5 Discussion 10

3 Theoretical Framework **11**
- 3.1 Introduction 11
- 3.2 Model Hamiltonian 11
- 3.3 Time Evolution and Bogoliubov Transformations 12
- 3.4 Covariance Matrix and Output State 13
- 3.5 Cluster States and Nullifiers 17
  - 3.5.1 Nullifiers and Adjacency Matrix Representation 17
  - 3.5.2 Covariance-Based Characterization and Connection to the Bogoliubov Formalism 17
  - 3.5.3 Performance Metrics 19
- 3.6 Losses 20

4 Reference State Protocol and Covariance Matrix Reconstruction **22**
- 4.1 Scaled Covariance Matrix 22
- 4.2 System Gain Calibration 23
- 4.3 Uncertainty budget 23
- 4.4 JTWPA gain estimation 24
- 4.5 Gaussianity Validation 24

4.6 Multiplexed Heterodyne Detection 25
4.7 Optimization Strategy: Maximizing Inter-mode Covariances 26
4.8 Reconstruction of the Covariance Matrix and Comparison with Theoretical Model 27
4.9 Discussion 29

A Examples of Covariance Matrices Analytical Calculations **31**
A.1 Quadrature Rotation 31
A.2 Linear Four-Mode Cluster with Distributed Ancillary Coupling: Covariance Matrix 32
A.3 Cyclic Four-Mode Cluster State with Ancillary Modes: Covariance Matrix . 33
A.4 Star Four-Mode Cluster State with Ancillary Modes: Covariance Matrix . . 33
A.5 Fully Connected Four-Mode Cluster State with Ancillary Modes: Covariance Matrix 34

List of Symbols

α_k Coherent amplitude of the k-th pump tone. See equation (S8), p. 12.

$\boldsymbol{\Delta}$ Nullifier covariance matrix. See equation (S37), p. 17.

$\boldsymbol{\Delta}_{\text{loss}}$ Nullifier covariance matrix accounting for the losses. . See equation (S51), p. 20.

$\boldsymbol{\Gamma}$ Reconstructed output covariance matrix. See equation (S54), p. 22.

$\boldsymbol{\Gamma}_\theta$ Rotated reconstructed output covariance matrix. See equation (S58), p. 23.

$\boldsymbol{\sigma_\theta}$ Rotated output covariance matrix . See equation (S66), p. 32.

$\boldsymbol{\sigma}$ Output covariance matrix. See equation (S25), p. 14.

$\boldsymbol{\sigma}_{\text{in}}$ Input state covariance matrix. See equation (S23), p. 14.

$\boldsymbol{\sigma}_{\text{vac}}$ Vacuum state covariance matrix. See equation (S24), p. 14.

$\boldsymbol{\theta}_{\text{opt}}$ Vector of angles which maximize $\mathcal{C}_{px}(\theta)$. See equation (S62), p. 28.

$\boldsymbol{M}$ Measured quadratures vector in photon basis. See equation (S57), p. 22.

$\boldsymbol{X_\theta}$ Rotated vector of quadrature operators. See equation (S59), p. 23.

$\boldsymbol{\sigma}_{\text{loss}}$ Output covariance matrix accounting for the losses . See equation (S49), p. 20.

$\boldsymbol{D}$ Losses matrix . See equation (S50), p. 20.

η_i Loss of the i-th mode at the JTWPA output . See equation (S48), p. 20.

$\hat{\boldsymbol{\delta}}$ Vector of the nullifier operators. See equation (S36), p. 17.

$\hat{\boldsymbol{A}}$ Vector of annihilation and creation operators. See equation (S10), p. 12.

$\hat{\boldsymbol{A}}_{\text{out}}$ Vector of output operators after time evolution. See equation (S10), p. 12.

$\hat{\boldsymbol{X}}$ Vector of quadrature operators . See equation (S19), p. 13.

$\hat{a}_{\text{out},i}^{(\text{loss})}$ Annihilation operator for the i-th mode at the JTWPA output accounting for the losses . See equation (S48), p. 20.

$\hat{a}_j$ Annihilation operator for the j-th mode. See equation (S4), p. 11.

$\hat{a}_j^\dagger$ Creation operator for the j-th mode. See equation (S4), p. 11.

$\hat{a}_{\text{out},i}$ Annihilation operator for the i-th mode at the JTWPA output . See equation (S13), p. 12.

$\hat{H}(t)$ Total Hamiltonian in the lab frame. See equation (S6), p. 11.

$\hat{H}_0$ Free Hamiltonian of the bosonic modes. See equation (S4), p. 11.

$\hat{H}_{\text{int}}(t)$ Interaction Hamiltonian in the lab frame. See equation (S5), p. 11.

$\hat{H}_I(t)$ Interaction Hamiltonian in the interaction picture respect to $\hat{H}_0$. See equation (S8), p. 12.

$\hat{I}_j$ j-th mode measured position quadrature operator. See equation (S55), p. 22.

$\hat{p}_j$ Momentum quadrature of mode j. See equation (S20), p. 13.

$\hat{Q}_j$ j-th mode measured momentum quadrature operator. See equation (S55), p. 22.

$\hat{U}_I(t)$ Time evolution operator in the interaction picture. See equation (S9), p. 12.

$\hat{x}_j$ Position quadrature of mode j. See equation (S20), p. 13.

$\langle\hat{\delta}^2\rangle_{\text{avg}}^{(\text{norm})}$ Normalized average nullifier variance. See equation (S46), p. 19.

$\mathbb{I}_{dim}$ Identity matrix of size *dim*.

$\mathbf{A}$ Binary adjacency matrix, p. 16.

$\mathbf{e}_k$ Canonical (standard) basis column vector with a 1 in the k-th position and 0 elsewhere. See equation (S65), p. 31.

$\mathbf{N}$ Nullifier matrix acting on the quadrature vector. See equation (S36), p. 17.

$\mathbf{P}$ Permutation matrix from block-wise to mode-wise notation . See equation (S65), p. 31.

$\mathbf{S}$ Symplectic matrix describing the Bogoliubov transformation. See equation (S11), p. 12.

$\mathbf{S}_X$ Symplectic matrix acting on quadrature space. See equation (S23), p. 14.

$\mathbf{U}$ Submatrix of $\mathbf{S}$ is the local squeezing matrix. See equation (S12), p. 12.

$\mathbf{V}$ Submatrix of $\mathbf{S}$ is the inter-mode squeezing matrix. See equation (S12), p. 12.

$\mathcal{C}_{px}(\theta)$ Mean value x-p correlations. See equation (S62), p. 26.

$\mathcal{T}$ Time-ordering operator. See equation (S9), p. 12.

ω_j Frequency of the j-th bosonic mode. See equation (S1), p. 9.

$\Omega_{jl}^{(k)}$	Frequency of the k-th pump tone. See equation (S5), p. 11.
ϕ_k	Phase of the k-th pump tone. See equation (S8), p. 12.
τ	Acquisition time. See equation (S56), p. 22.
$\mathbf{R}_\theta$	Rotation matrix. See equation (S59), p. 23.
$\mathbf{\Gamma}^{\text{exp}}$	Scaled measured covariance matrix. See equation (S57), p. 22.
$\mathbf{G}$	Matrix generator associated with the effective Hamiltonian. See equation (S18), p. 13.
$g_{jl}^{(k)}$	Nonlinear coupling coefficient between modes j and l under pump k. See equation (S5), p. 11.
$G_{\text{SYS},j}$	j-th mode system gain. See equation (S55), p. 22.
G_j	j-th mode JTWPA gain . See equation (S61), p. 24.
$I_j^{(\text{raw})}$	j-th mode measured voltage position quadrature. See equation (S56), p. 22.
i_j	j-th mode measured position quadrature in photon-number units. See equation (S56), p. 22.
m	Total number of bosonic modes. See equation (S4), p. 11.
n	Number of classical pump tones. See equation (S5), p. 11.
$Q_j^{(\text{raw})}$	j-th mode measured voltage momentum quadrature. See equation (S56), p. 22.
q_j	j-th mode measured momentum quadrature in photon-number units. See equation (S56), p. 22.
$r_{jl}^{(k)}$	Effective squeezing parameter from pump k between modes j and l. See equation (S14), p. 13.
$T_{\text{SYS},j}$	j-th mode system noise temperature . See equation (S60), p. 23.
T_{VTS}	Variable Temperature Stage (VTS) temperature. See equation (S60), p. 23.
Z_0	Characteristic impedance of the transmission line. See equation (S56), p. 22.
S_{F}	Frobenius similarity. See equation (S47), p. 20.
$\mathbf{J}$	Symplectic form. See equation (S17), p. 13.

1 Detailed Experimental Scheme

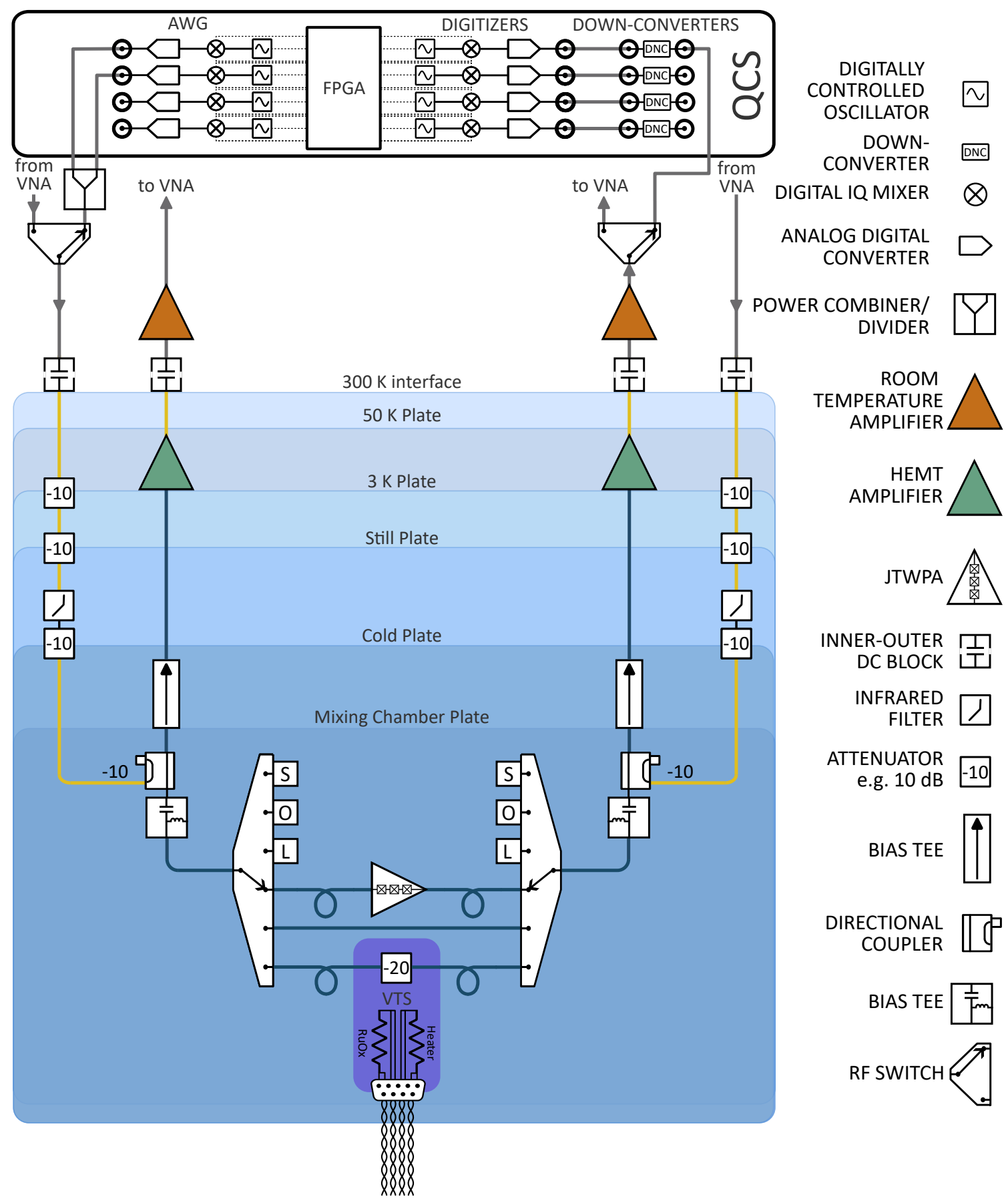

Supplementary Figure 1: **Schematic of the full cryogenic and room-temperature measurement setup.** The experiment is hosted in a dilution refrigerator, where two input lines are coupled to two output lines at the mixing chamber plate. Two cryogenic electromechanical switches allow for Short-Open-Load-Reciprocal (SOLR) deembedding and in situ noise calibration using a variable temperature stage (VTS) connected to a thermalized 20 dB attenuator [1, 2]. At room temperature, two channels of an Arbitrary Waveform Generator (AWG) are used for pump generation, while a channel of a downconverter (DNC) and a digitizer (DIG) is used for heterodyne detection. The AWG, DNC, and DIG are components of a digital Quantum Control System (QCS) (Keysight Technologies, Santa Rosa, CA, USA). Two additional switches allow routing to a vector network analyzer (VNA) for preliminary characterization [3].

2 JTWPA Details and Pumps and Modes Schemes Selection

2.1 JTWPA details

The device investigated in this study is a pre-commercial AI-TWPA-C [4] from Arctic Instruments, featuring a central conductor composed of a series array of flux-tunable SNAILs (Superconducting Nonlinear Asymmetric Inductive eLements) (see Supplementary Figure 2) [5]. A uniform DC flux applied through an on-chip bias line maximizes three-wave mixing (3WM), while leaving a residual four-wave mixing (4WM) component and, consequently, a Kerr effect. Periodically integrated spiral resonators and capacitors are used to engineer the line dispersion, producing a superlinear response in the frequency range of the second pump harmonic under typical operating conditions. This makes its direct generation inefficient, yet sufficient for back-conversion to the pump, which imparts a controlled phase shift (effective Kerr effect) essential for maintaining broadband phase matching and gain. The AI-TWPA-C is fabricated using multilayer niobium Josephson technology, with Nb/Al–AlOx/Nb trilayers forming the junctions. The primary niobium layer constructs the CPW ground planes and capacitors, while a secondary niobium layer carries the meandering flux-bias line and the top electrodes of the shunt capacitors. Thin normal-metal films define resistive terminations to ground, stabilizing the flux bias.

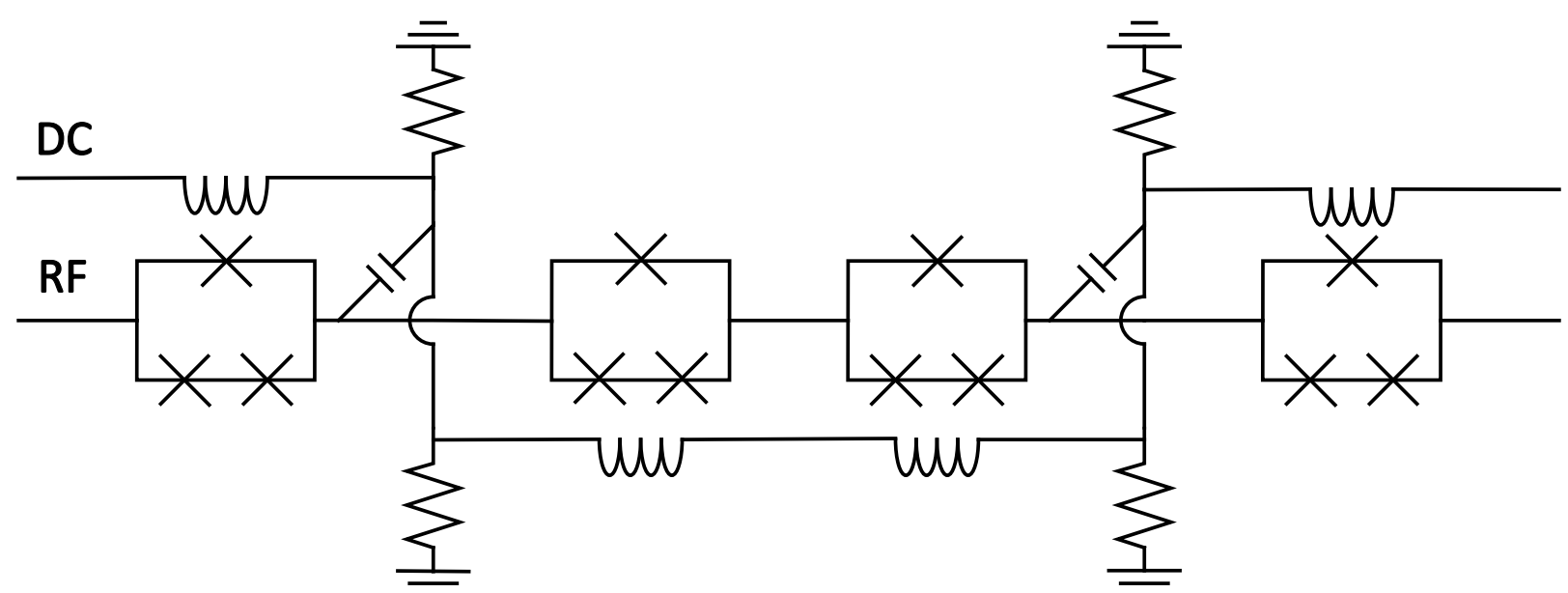

Supplementary Figure 2: **Circuit diagram of the JTWPA, adapted from Perelshtein, 2022 [4].**

2.2 JTWPA Gain Flatness

To ensure optimal entanglement conditions for the generation of continuous-variable cluster states, particular care was devoted to the positioning of the pump tones and frequency modes with respect to the transmission of the Josephson Traveling Wave Parametric Amplifier (JTWPA). Supplementary Figure 3 displays the transmission of the JTWPA chip, embedded in its packaging and connected to electromechanical switches via uncompensated cables [2], as measured across all pump frequencies used in this experiment. In the frequency range of interest, centered around $\omega = 2\pi \cdot 4.5$ GHz, the amplifier provides a

sufficiently flat gain profile for all the generated pump configurations. This behavior guarantees minimal amplitude distortion and phase dispersion, which are critical for preserving the entanglement structure of the generated cluster states.

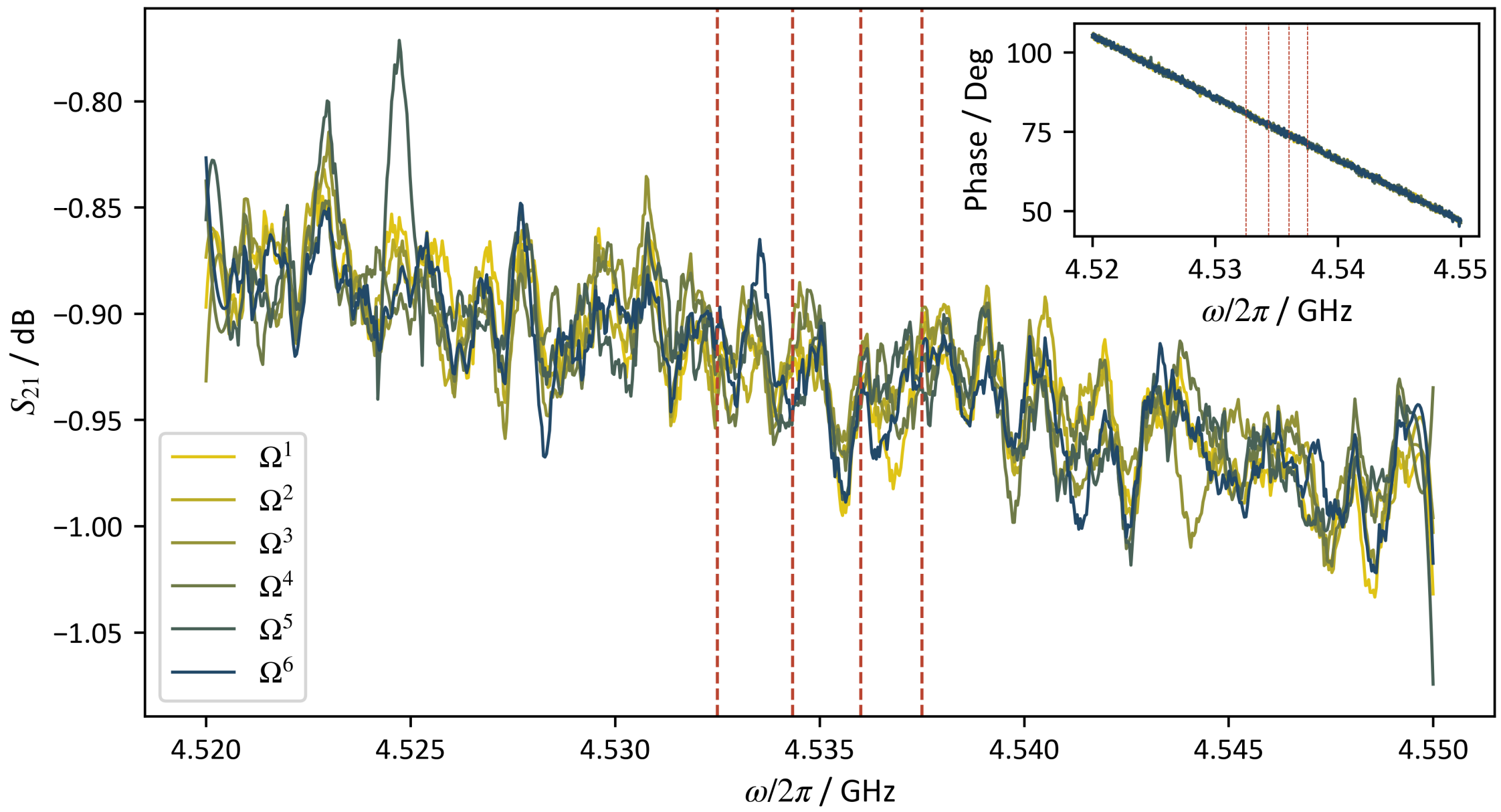

Supplementary Figure 3: **Josephson Traveling-Wave Parametric Amplifier: Preliminary Characterization.** Transmission coefficient S_{21} of the device under test (DUT) as a function of signal frequency, measured for six pump frequencies $\Omega^{(k)}$ at fixed pump power $P_\Omega = -21\,\text{dBm}$. The DUT consists of the JTWPA integrated in its package and connected to electromechanical switches through uncompensated cables. The S-parameters are referenced to the electromechanical switches and are obtained via a Short–Open–Load–Reciprocal (SOLR) calibration procedure. The amplifier is flux-biased at $\Phi = 0.378\,\Phi_0$ to operate predominantly in the three-wave mixing regime. Dashed vertical lines mark the resonant modes of the investigated cluster. The inset displays the phase of the transmission coefficient S_{21}.

2.3 JTWPA two-mode squeezing generation

The device under test was characterized in terms of variance of nullifier with a simplified graph composed of only two connected nodes, which coincide with the familiar two-mode squeezing operator. The selected nodes here were the edges of our main cluster states modes, $\omega_1 = 2\pi \cdot 4.5325$ GHz and $\omega_4 = 2\pi \cdot 4.5375$ GHz. The variance of the nullifier, plotted in Supplementary Figure 4 as a function of the pump power, are consistent with

the one obtained in Reference [4].

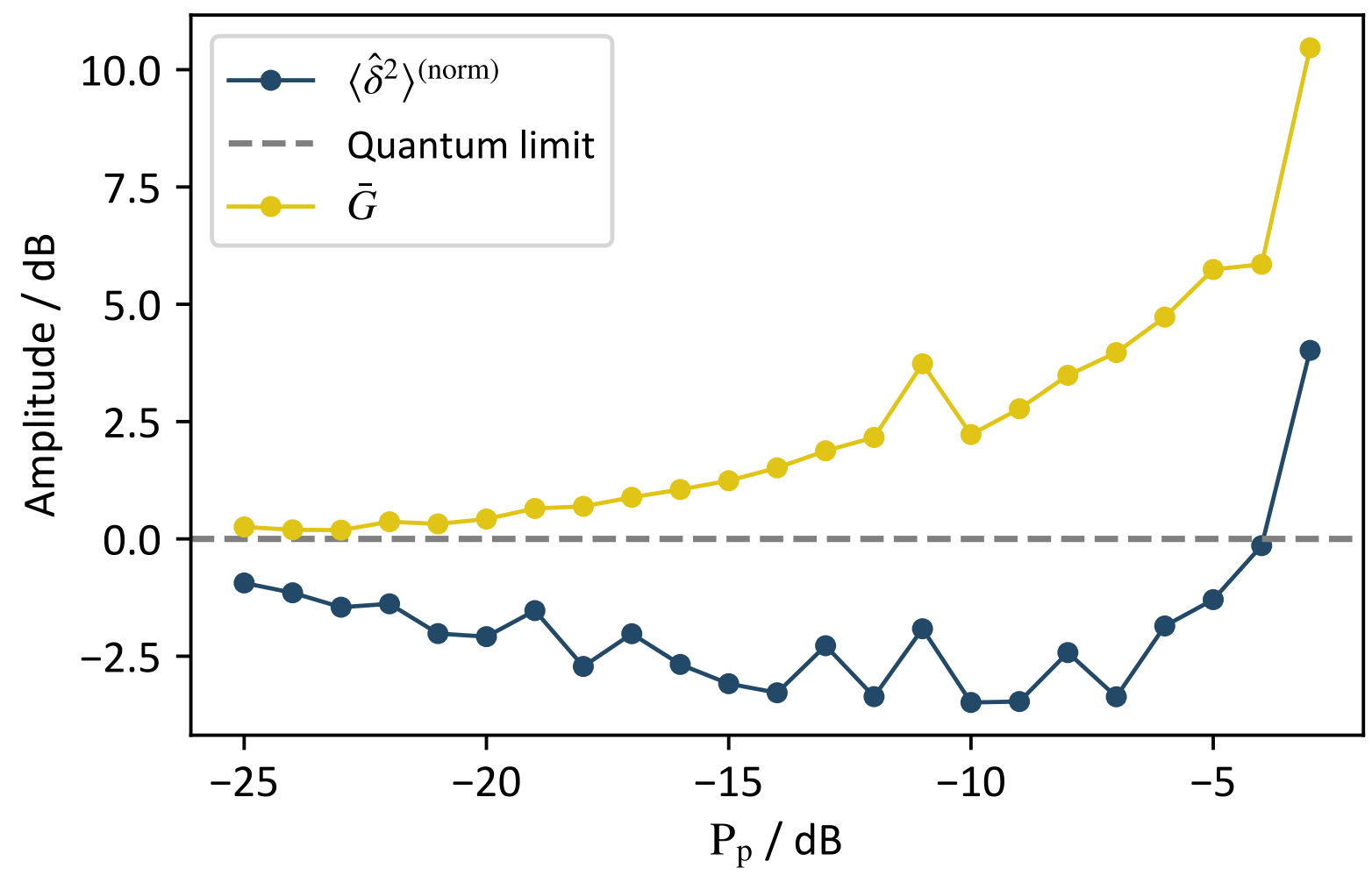

Supplementary Figure 4: **Two-mode squeezing for different pump powers.** Two-mode squeezing generation $\langle\hat{\delta}^2\rangle^{(\mathrm{norm})}$ and mean JTWPA gain $\bar{G}$ at the frequencies of interest, $\omega_1 = 2\pi \cdot 4.5325$ GHz and $\omega_4 = 2\pi \cdot 4.5375$ GHz, as a function of the pump power.

2.4 Spectral Engineering for Cluster State Generation

To study the impact of spectral arrangement on the distribution of entanglement in the generated cluster states, we engineered the mode frequency distribution using a sinusoidally modulated spacing:

$$\omega_j = \omega_{\mathrm{start}} + (j-1)\left(\frac{\omega_{\mathrm{start}} - \omega_{\mathrm{end}}}{N-1}\right) + A\sin\left(\frac{\pi(j-1)}{N-1}\right), \qquad j = 1, \ldots, N, \tag{S1}$$

where ω_{start} and ω_{end} are the frequency edges of the cluster state, A is the modulation amplitude, and N is the total number of modes.

Choosing $\omega_{\mathrm{start}} = 2\pi \cdot 4.5325$ GHz, $\omega_{\mathrm{end}} = 2\pi \cdot 4.5375$ GHz, $A = 2\pi \cdot 0.2$ MHz, and $N = 4$:

$$\begin{aligned}
\omega_1 &= 2\pi \cdot 4.5325 \text{ GHz},\\
\omega_2 &= 2\pi \cdot 4.5343 \text{ GHz},\\
\omega_3 &= 2\pi \cdot 4.5360 \text{ GHz},\\
\omega_4 &= 2\pi \cdot 4.5375 \text{ GHz}.
\end{aligned} \tag{S2}$$

Pump tones were designed by computing all pairwise interactions using:

$$\Omega_{jl}^{(k)} = \omega_j + \omega_l, \tag{S3}$$

for each pair (j, l) defined by the target cluster topology.

The sinusoidal modulation plays a critical role in cluster state generation. Specifically, it prevents degeneracies in the pump tone frequencies which are used to establish pairwise entanglement. Without modulation, uniform mode spacing can cause multiple pairs to share identical Ω_{jl} values, leading to ambiguous interaction topologies and reduced control over the entanglement graph. By introducing the sinusoidal term, each Ω_{jl} becomes distinct, enabling precise and unambiguous implementation of the desired cluster connectivity.

The supported entanglement graph configurations include:

- **Linear:** Nearest-neighbor connections.
- **Star:** A central hub mode connected to all others.
- **Cyclic:** Ring topology with periodic boundary conditions.
- **Fully Connected:** All-to-all entanglement.

2.5 Discussion

The combination of flat JTWPA gain in the targeted frequency band and the systematic generation of pump-mode configurations allows us to synthesize a variety of cluster-state topologies. These results validate the suitability of the amplifier for scalable continuous-variable quantum computing platforms.

3 Theoretical Framework

3.1 Introduction

Nonlinear media driven by multiple coherent pump tones enable the generation of multimode entangled states via parametric interactions. Such platforms are central to continuous-variable (CV) quantum information processing, and are particularly relevant for the generation of Gaussian cluster states used in measurement-based quantum computing.

In this work, we examine the most general scenario of m bosonic modes coupled through n pump tones, each characterized by arbitrary amplitude, frequency, and phase. We investigate the resulting squeezing properties and mode correlations by analyzing the output covariance matrix and introducing the concept of a cluster nullifier.

3.2 Model Hamiltonian

We consider a bosonic system composed of m modes described by annihilation operators $\hat{a}_j$ with free Hamiltonian

$$\hat{H}_0 = \sum_{j=1}^{m} \omega_j \hat{a}_j^\dagger \hat{a}_j, \tag{S4}$$

where ω_j is the frequency of the j-th mode.

The system is driven by n classical pump tones, each described by frequency $\Omega_{jl}^{(k)}$, amplitude α_k, and phase ϕ_k. These tones induce three-wave mixing interactions via a second-order nonlinearity.

The interaction Hamiltonian in the rotating wave approximation reads

$$\hat{H}_{\text{int}}(t) = \sum_{k=1}^{n} \sum_{j \le l} g_{jl}^{(k)} \alpha_k e^{-i\Omega_{jl}^{(k)} t - i\phi_k} \hat{a}_j^\dagger \hat{a}_l^\dagger + \text{H.c.}, \tag{S5}$$

where $g_{jl}^{(k)} \in \mathbb{C}$ encodes the nonlinear coupling between modes j and l under pump k. Symmetry $g_{jl}^{(k)} = g_{lj}^{(k)}$ is assumed. The total Hamiltonian is

$$\hat{H}(t) = \hat{H}_0 + \hat{H}_{\text{int}}(t). \tag{S6}$$

In the interaction picture with respect to $\hat{H}_0$, the bosonic operators evolve as

$$\hat{a}_j(t) = \hat{a}_j e^{-i\omega_j t}, \tag{S7}$$

and the interaction Hamiltonian becomes

$$\hat{H}_I(t) = \sum_{k=1}^{n} \sum_{j \le l} g_{jl}^{(k)} \alpha_k e^{-i(\Omega_{jl}^{(k)} - \omega_j - \omega_l)t - i\phi_k} \hat{a}_j^\dagger \hat{a}_l^\dagger + \text{H.c.} \tag{S8}$$

The resonance condition $\Omega_{jl}^{(k)} \approx \omega_j + \omega_l$ selects the relevant processes contributing to pair creation in modes j and l. Each tone drives a specific Bogoliubov transformation depending on its frequency and phase.

3.3 Time Evolution and Bogoliubov Transformations

We work in the interaction picture defined by the free Hamiltonian $\hat{H}_0$. The time evolution operator in this picture is

$$\hat{U}_I(t) = \mathcal{T} \exp\left(-i \int_0^t \hat{H}_I(t')\mathrm{d}t'\right), \tag{S9}$$

where $\mathcal{T}$ denotes time-ordering. In the case of weak coupling or short times, we can approximate the evolution as Gaussian and neglect higher-order time-ordering effects. The effective transformation of mode operators is linear and described by a Bogoliubov transformation.

Let us define the vector of annihilation and creation operators $\hat{\boldsymbol{A}}$ and the vector of output operators after time evolution $\hat{\boldsymbol{A}}_{\text{out}}$:

$$\hat{\boldsymbol{A}} = \left(\hat{a}_1, \cdots, \hat{a}_m, \hat{a}_1^\dagger, \cdots, \hat{a}_m^\dagger\right)^\top, \quad \hat{\boldsymbol{A}}_{\text{out}} = \hat{U}_I^\dagger(t)\, \hat{\boldsymbol{A}}\, \hat{U}_I(t). \tag{S10}$$

This time evolution implements a symplectic transformation:

$$\hat{\boldsymbol{A}}_{\text{out}} = \mathbf{S}\hat{\boldsymbol{A}}, \tag{S11}$$

where the symplectic matrix

$\mathbf{S} \in \mathrm{Sp}(2m, \mathbb{R})$ takes the form:

$$\mathbf{S} = \begin{pmatrix} \mathbf{U} & \mathbf{V} \\ \mathbf{V}^* & \mathbf{U}^* \end{pmatrix}, \quad \text{with } \mathbf{U}\mathbf{U}^\dagger - \mathbf{V}\mathbf{V}^\dagger = \mathbb{I}, \tag{S12}$$

recovering the matrix form of the common input-output relation

$$\hat{a}_{\text{out},i} = \sum_j \left(\mathbf{U}_{ij} a_i + \mathbf{V}_{ij} \hat{a}_j^\dagger\right). \tag{S13}$$

The Bogoliubov matrices $\mathbf{U}$ and $\mathbf{V}$ describe how a Gaussian unitary transformation mixes the input bosonic modes. $\mathbf{U}$ is the local squeezing matrix and captures linear mode mixing, while $\mathbf{V}$ is the inter-mode squeezing matrix and captures squeezing and pair creation effects.

In the regime where each tone k is near-resonant with a pair $(\omega_j + \omega_l)$, the corresponding contribution to the interaction Hamiltonian is approximately time-independent in the rotating frame. We can then write the total Hamiltonian as:

$$\hat{H}_{\text{eff}} = \sum_{k=1}^{n} \sum_{j \leq l} \left(r_{jl}^{(k)} \hat{a}_j^\dagger \hat{a}_l^\dagger + r_{jl}^{(k)*} \hat{a}_j \hat{a}_l \right), \tag{S14}$$

where we define the effective squeezing parameters as:

$$r_{jl}^{(k)} = g_{jl}^{(k)} \alpha_k e^{-i\phi_k}. \tag{S15}$$

The total Hamiltonian is quadratic in the field operators and generates a Gaussian transformation. Matrix $\mathbf{V}$ is directly related to the sum of all $r_{jl}^{(k)}$, allowing arbitrary interference between multiple pump tones with different phases.

The resulting symplectic transformation can be computed via matrix exponentiation:

$$\mathbf{S}(t) = \exp\left(\mathbf{J} \cdot \mathbf{G} \cdot t\right), \tag{S16}$$

where

$$\mathbf{J} = \begin{pmatrix} \mathbf{0}_m & \mathbb{I}_m \\ -\mathbb{I}_m & \mathbf{0}_m \end{pmatrix} \tag{S17}$$

is the symplectic form and

$$\mathbf{G} \ = \ \begin{pmatrix} 0 & \mathbf{G} \\ \mathbf{G}^* & 0 \end{pmatrix}, \quad (\mathbf{G})_{jl} \ = \ \sum_{k=1}^{n} r_{jl}^{(k)} \tag{S18}$$

is the generator associated with $\hat{H}_{\text{eff}}$.

$\mathbf{S}(t)$ maps the vector of input operators to that of the output operators, preserving all commutation relations. From $\mathbf{S}(t)$ one directly extracts the squeezing parameters and inter-mode correlations, enabling both analytical and numerical evaluation of the output state's squeezing and entanglement properties.

3.4 Covariance Matrix and Output State

To characterize the quantum state of the output field, we define the vector of quadrature operators in block-wise order:

$$\hat{\boldsymbol{X}} = \left(\hat{x}_1, \cdots, \hat{x}_m, \hat{p}_1, \cdots, \hat{p}_m\right)^\top \tag{S19}$$

where

$$\hat{x}_j = \frac{1}{\sqrt{2}} \left(\hat{a}_j + \hat{a}_j^\dagger\right), \quad \hat{p}_j = \frac{1}{\sqrt{2}i} \left(\hat{a}_j - \hat{a}_j^\dagger\right). \tag{S20}$$

These quadratures obey the block-wise commutation relations:

$$[\hat{X}_j, \hat{X}_l] = i\mathrm{J}_{\mathrm{jl}}. \tag{S21}$$

where J_{jl} are the elements of the symplectic matrix defined in Eq. S17.

The state of the system is fully characterized by the first moments $\langle \hat{\boldsymbol{X}} \rangle$ and the covariance matrix $\boldsymbol{\sigma}$, defined by:

$$\sigma_{jl} = \frac{1}{2}\langle \hat{X}_j \hat{X}_l + \hat{X}_l \hat{X}_j \rangle - \langle \hat{X}_j \rangle \langle \hat{X}_l \rangle. \tag{S22}$$

For a quadratic Hamiltonian and an initial vacuum state (a Gaussian state), the system evolves within the space of Gaussian states. The time-evolution operator $\hat{U}_I(t)$ induces a symplectic transformation:

$$\hat{\boldsymbol{X}}_{\mathrm{out}} = \mathbf{S}_X \hat{\boldsymbol{X}}, \qquad \boldsymbol{\sigma} = \mathbf{S}_X \boldsymbol{\sigma}_{\mathrm{in}} \mathbf{S}_X^{\top}, \tag{S23}$$

where $\mathbf{S}_X$ is a real symplectic matrix acting on quadrature space and $\boldsymbol{\sigma}_{\mathrm{in}}$ is the input state covariance matrix. For the vacuum input:

$$\boldsymbol{\sigma}_{\mathrm{in}} = \boldsymbol{\sigma}_{\mathrm{vac}} = \frac{1}{2}\mathbb{I}_{2m}. \tag{S24}$$

Therefore, the output covariance matrix becomes:

$$\boldsymbol{\sigma} = \frac{1}{2}\mathbf{S}_X \mathbf{S}_X^{\top}. \tag{S25}$$

The quadrature operators are related to the mode operators via the linear transformation:

$$\hat{\boldsymbol{X}} = \mathbf{T}\hat{\boldsymbol{A}}, \qquad \mathbf{T} = \frac{1}{\sqrt{2}}\begin{pmatrix} \mathbb{I}_m & -i\mathbb{I}_m \\ \mathbb{I}_m & i\mathbb{I}_m \end{pmatrix}. \tag{S26}$$

Given the Bogoliubov transformation acting on mode operators, defined in Eq. (S11) and Eq. (S12), the induced symplectic transformation in block-wise space is:

$$\mathbf{S}_X = \mathbf{T}\,\mathbf{S}\,\mathbf{T}^{-1}. \tag{S27}$$

Carrying out this matrix product yields:

$$\mathbf{S}_X = \begin{pmatrix} \mathrm{Re}(\mathbf{U}+\mathbf{V}) & -\mathrm{Im}(\mathbf{U}-\mathbf{V}) \\ \mathrm{Im}(\mathbf{U}+\mathbf{V}) & \mathrm{Re}(\mathbf{U}-\mathbf{V}) \end{pmatrix}, \tag{S28}$$

where all blocks are $m \times m$ real matrices.

Substituting into Eq. (S25), the final expression for the output covariance matrix is:

$$\boldsymbol{\sigma} = \frac{1}{2}\begin{pmatrix} \mathrm{Re}(\mathbf{U}+\mathbf{V}) & -\mathrm{Im}(\mathbf{U}-\mathbf{V}) \\ \mathrm{Im}(\mathbf{U}+\mathbf{V}) & \mathrm{Re}(\mathbf{U}-\mathbf{V}) \end{pmatrix}\begin{pmatrix} \mathrm{Re}(\mathbf{U}+\mathbf{V})^{\top} & \mathrm{Im}(\mathbf{U}+\mathbf{V})^{\top} \\ -\mathrm{Im}(\mathbf{U}-\mathbf{V})^{\top} & \mathrm{Re}(\mathbf{U}-\mathbf{V})^{\top} \end{pmatrix}. \tag{S29}$$

This real, symmetric $2m \times 2m$ matrix fully describes the Gaussian output state in mode-wise order and is directly compatible with the standard symplectic structure used in continuous-variable quantum information protocols.

Assumption 1: Uniform Single-Mode Squeezing. We consider a configuration in which each mode of a multimode transmission line undergoes identical, uncorrelated single-mode squeezing. This can be approximately realized using a multi-pump traveling-wave parametric amplifier (TWPA), where each mode is driven by a separate pump tone tuned to twice its frequency (degenerate parametric amplification). To ensure uniform squeezing across all modes, the following conditions must be met:

- a flat gain profile across the operational bandwidth,
- negligible dispersion along the transmission line,
- stable phase coherence among the pump tones.

These engineering requirements can be satisfied via spectral pump shaping and precise calibration of the transmission line properties.

Under these conditions, all modes experience equal squeezing strength, defined by the modulus of the squeezing coefficient $|r|$ in Eq. S14, and aligned squeezing angles. In this regime, the Bogoliubov matrices take a simple diagonal form:

$$\mathbf{U} = \cosh|r| \cdot \mathbb{I}_m, \qquad \mathbf{V} = \sinh|r| \cdot \mathbb{I}_m, \tag{S30}$$

Each (x_j, p_j) pair is independently squeezed. The variances are:

$$\mathrm{Var}(x_j) = \frac{1}{2}e^{-2|r|}, \qquad \mathrm{Var}(p_j) = \frac{1}{2}e^{2|r|}. \tag{S31}$$

Assumption 2: Pure Two-Mode Squeezing Between Arbitrary Modes. We now consider ideal two-mode squeezing between an arbitrary pair of modes j and k (with $j \neq k$), while all other modes remain in vacuum. This can be realized in a multi-pumped TWPA architecture by applying a pump tone at frequency $\Omega_{jk} = \omega_j + \omega_k$, enabling non-degenerate parametric down-conversion via three-wave mixing. If coupling to other modes is negligible—achieved through spectral sparsity, dispersion engineering, and pump frequency selection—the effective interaction is confined to the selected pair (j, k).

To realize clean, isolated two-mode squeezing in practice, several physical conditions must be satisfied:

- **Spectral selectivity:** The mode spectrum should be sufficiently sparse or engineered to avoid accidental degeneracies, ensuring that each pump addresses only its intended mode pair.
- **Minimal dispersion:** The transmission line should exhibit low dispersion across the operating bandwidth to maintain consistent phase matching across the selected frequency pairs.

- **Pump coherence and stability:** The pump tones must be phase-stable and frequency-precise to prevent drift or cross-coupling between unintended modes.

These requirements can be addressed through careful design of the TWPA geometry, spectral shaping of the pump distribution, and precise calibration of system parameters. When these conditions are met, the squeezing interaction is effectively localized to the selected mode pair, and the squeezing strengh $|r|$ can be assumed uniform and the squeezing angles aligned across all pairs.

Under these assumptions, the Bogoliubov matrices $\mathbf{U}$ and $\mathbf{V}$ acquire a block structure determined by the mode connectivity. To capture this structure explicitly, we define the symmetric binary adjacency matrix $\mathbf{A} \in \{0,1\}^{m\times m}$. For a pure two-mode squeezed state coupling only modes j and l, the matrix $\mathbf{A}$ is zero everywhere except:

$$\mathbf{A}_{jl} = \mathbf{A}_{lj} = 1.$$

The Bogoliubov matrices become:

$$\mathbf{U} = \mathbb{I}_m + (\cosh|r| - 1)\cdot \mathrm{diag}(\mathbf{D}), \qquad \mathbf{V} = \sinh|r|\cdot \mathbf{A}, \tag{S32}$$

where $\mathbf{D}$ is a diagonal matrix encoding mode participation in two-mode squeezing processes. Specifically, $\mathbf{D}_{jj} = 1$ if mode j is coupled to at least one other mode via a nonzero entry in $\mathbf{A}$, and $\mathbf{D}_{jj} = 0$ otherwise. That is,

$$\mathbf{D}_{jj} = \begin{cases} 1, & \text{if } \sum_{l=1}^{m} \mathbf{A}_{jl} > 0, \\ 0, & \text{otherwise.} \end{cases}$$

This construction ensures that each mode involved in squeezing undergoes the correct Bogoliubov transformation gain in $\mathbf{U}$, while modes that are not coupled remain unaffected.

In the simple case of a single pair (j,l), the matrix $\mathbf{A}$ has value 1 at (j,l) and (l,j), and zeros elsewhere. This formulation generalizes naturally to arbitrary graphs of squeezed pairs: any symmetric adjacency matrix $\mathbf{A}$ with entries in $\{0,1\}$ encodes which modes are pairwise entangled via two-mode squeezing. The resulting matrices $\mathbf{U}$ and $\mathbf{V}$ then describe a multimode Gaussian state with an entanglement structure.

These matrices are real and symmetric, implying:

$$\mathrm{Im}(\mathbf{U} \pm \mathbf{V}) = 0, \qquad \mathrm{Re}(\mathbf{U} \pm \mathbf{V}) = \mathbf{U} \pm \mathbf{V}.$$

Constructing the real symplectic transformation $\mathbf{S}_X$ in mode-wise quadrature order:

$$\mathbf{S}_X = \begin{pmatrix} \mathbf{U}+\mathbf{V} & 0 \\ 0 & \mathbf{U}-\mathbf{V} \end{pmatrix}, \tag{S33}$$

we find the output covariance matrix as:

$$\boldsymbol{\sigma} = \frac{1}{2}\mathbf{S}_X\,\mathbf{S}_X^\top = \frac{1}{2}\begin{pmatrix} (\mathbf{U}+\mathbf{V})^2 & 0 \\ 0 & (\mathbf{U}-\mathbf{V})^2 \end{pmatrix}. \tag{S34}$$

3.5 Cluster States and Nullifiers

Cluster states are a central resource for continuous-variable (CV) measurement-based quantum computation. These multipartite entangled Gaussian states are defined, in the infinite squeezing limit, as simultaneous zero-eigenstates of a set of commuting observables called *nullifiers*. They generalize the stabilizer formalism of discrete-variable cluster states to the CV setting.

3.5.1 Nullifiers and Adjacency Matrix Representation

A CV cluster state associated with a symmetric adjacency matrix $\mathbf{A}$ is ideally characterized by the vanishing of the nullifier operators. For a given cluster state defined by an adjacency matrix, the ideal nullifier operator associated with node j takes the form:

$$\hat{\delta}_j = \hat{p}_j - \sum_{l=1}^{m} A_{jl}\,\hat{x}_l, \qquad j = 1, \ldots, m. \tag{S35}$$

where $\hat{x}_l$ and $\hat{p}_j$ are the position and momentum quadratures of the modes. The nullifier operator couples the momentum quadrature of a given mode to the position quadratures of its graph neighbors, thereby reflecting the entanglement structure imposed by the graph topology.

In the block-wise quadrature basis, the nullifier vector takes the form:

$$\hat{\boldsymbol{\delta}} = \mathbf{N} \cdot \hat{\boldsymbol{X}}, \qquad \text{with} \quad \mathbf{N} = \left(-\mathbf{A} \mid \mathbb{I}_m\right). \tag{S36}$$

This definition naturally encodes the entanglement structure of the target cluster state. In the ideal limit of infinite squeezing, the variances of the nullifiers vanish, and the state becomes fully supported on the common eigenspace of the nullifiers.

3.5.2 Covariance-Based Characterization and Connection to the Bogoliubov Formalism

Let $\boldsymbol{\sigma} \in \mathbb{R}^{2m\times 2m}$ denote the covariance matrix of the output Gaussian state. The noise in the nullifier observables is quantified by the nullifier covariance matrix:

$$\boldsymbol{\Delta} = \mathbf{N}\boldsymbol{\sigma}\mathbf{N}^{\top}, \tag{S37}$$

where each diagonal entry Δ_{jj} corresponds to the variance of the nullifier operator $\hat{\delta}_j$, i.e.,

$$\Delta_{jj} = \langle \hat{\delta}_j^2 \rangle - \langle \hat{\delta}_j \rangle^2 = \mathrm{Var}(\hat{\delta}_j).$$

This directly quantifies how closely the physical state approximates the ideal cluster condition $\hat{\delta}_j = 0$, and allows a mode-by-mode assessment of the squeezing and entanglement structure. In particular, the eigenvalues of $\boldsymbol{\Delta}$ quantify the noise present in orthogonal linear

combinations of the nullifier operators. Smaller eigenvalues correspond to directions in nullifier space where the noise (i.e., variance) is minimized, indicating that the corresponding combinations are closer to ideal quantum correlations. Since $\mathbf{\Delta}$ is a real, symmetric, and positive semi-definite matrix, its eigenvalues can be interpreted as generalized variances along principal axes defined by a suitable basis transformation. This diagonalization effectively identifies the most and least squeezed (i.e., quantum) collective modes of the system. When all eigenvalues are significantly below the vacuum noise level (e.g., $1/2$ in units where $\hbar = 1$), the state exhibits genuine multipartite entanglement and closely approximates the ideal cluster state. Therefore, analyzing the spectrum of $\mathbf{\Delta}$ provides a basis-independent and physically meaningful method to assess the quantumness and fidelity of the generated state.

Example: Tripartite Cyclic Cluster State (Ideal Squeezing)

As a concrete example, consider a tripartite cluster state with a cyclic topology, where each of the three modes is connected to the other two. The corresponding binary adjacency matrix $\mathbf{A} \in \{0,1\}^{3\times 3}$ is:

$$\mathbf{A} = \begin{pmatrix} 0 & 1 & 1 \\ 1 & 0 & 1 \\ 1 & 1 & 0 \end{pmatrix}. \tag{S38}$$

The ideal nullifiers for this state take the form:

$$\hat{\delta}_1 = \hat{p}_1 - \hat{x}_2 - \hat{x}_3, \tag{S39}$$

$$\hat{\delta}_2 = \hat{p}_2 - \hat{x}_1 - \hat{x}_3, \tag{S40}$$

$$\hat{\delta}_3 = \hat{p}_3 - \hat{x}_1 - \hat{x}_2. \tag{S41}$$

In the infinite squeezing limit, an ideal cluster state satisfies

$$\hat{\delta}_j|\psi_{\text{cluster}}\rangle = 0 \qquad \text{for } j = 1, 2, 3. \tag{S42}$$

This means the quantum state $|\psi_{\text{cluster}}\rangle$ lies in the simultaneous zero-eigenspace of the three nullifiers. The vanishing of their variances,

$$\langle \hat{\delta}_j^2 \rangle \to 0 \quad \text{as} \quad |r| \to \infty, \tag{S43}$$

indicates perfect entanglement and full compliance with the cluster-state stabilizer conditions. In realistic scenarios with finite squeezing, these variances remain small but nonzero, and their values serve as fidelity indicators with respect to the ideal state.

In the case of pure two-mode squeezing, the matrices $\mathbf{U}$ and $\mathbf{V}$ were shown to be real and symmetric, with their structure determined by matrix $\mathbf{A}$, indicating physical connectivity:

$$\mathbf{V} = \sinh|r| \cdot \mathbf{A}. \tag{S44}$$

While $\mathbf{V}$ describes which mode pairs are entangled through squeezing operations, $\mathbf{A}$ defines the target cluster state graph in block-wise space. These matrices are related conceptually, but not necessarily equal: $\mathbf{V}$ encodes the actual implementation of squeezing interactions, while $\mathbf{A}$ describes the structure of nullifiers and desired stabilizers.

In optimized designs, one seeks to engineer $\mathbf{V}$ such that the resulting $\boldsymbol{\sigma}$ leads to nullifier variances that are minimized for a chosen $\mathbf{A}$. This can be accomplished by tuning the pump tones to induce a pattern of effective squeezing coefficients $r_{jl}^{(k)}$ that sum (via Eq. (S12)) into a $\mathbf{V}$ approximately proportional to $\mathbf{A}$, or via more general linear transformations.

3.5.3 Performance Metrics

To assess the quality of an approximate cluster state, one can compute the following figures of merit from the nullifier covariance matrix $\boldsymbol{\Delta}$, normalized with respect to the structure-dependent shot noise limit (SNL). This normalization enables fair comparison across different graph topologies defined by the adjacency matrix $\mathbf{A}$.

From the nullifier operators definition in Eq. (S35) and assuming that all modes are initialized in vacuum (i.e., $\langle \hat{x}_l^2 \rangle = \langle \hat{p}_j^2 \rangle = 1/2$, and all covariances vanish), the variance of the nullifier is:

$$\langle \hat{\delta}_j^2 \rangle = \langle \hat{p}_j^2 \rangle + \sum_l A_{jl}^2 \langle \hat{x}_l^2 \rangle = \frac{1}{2} \left(1 + \sum_l A_{jl}^2 \right). \tag{S45}$$

This expression captures how vacuum fluctuations propagate through the cluster state's graph structure. A similar derivation appears, for example, in the theoretical framework described by Menicucci et al. [6] and further discussed in Gaussian cluster-state literature (e.g., [7]).

Using this, we define the

- **normalized average nullifier variance**:

$$\langle \hat{\delta}^2 \rangle_{\text{avg}}^{(\text{norm})} = \frac{1}{m} \sum_{j=1}^{m} \frac{2\boldsymbol{\Delta}_{jj}}{1 + \sum_l A_{jl}^2}, \tag{S46}$$

which accounts for the vacuum noise scaling imposed by the cluster state's graph connectivity. The normalized variances directly indicate how much squeezing is achieved relative to the ideal limit, and values below 1 imply entanglement in the corresponding nullifier mode. Meanwhile, the Frobenius similarity provides a global, quantitative measure of state closeness, incorporating all quadrature correlations simultaneously.

This nullifier-based formalism thus bridges experimental observables (via $\mathbf{V}$ and $\boldsymbol{\sigma}$) with the graph-theoretic structure of ideal cluster states (encoded in $\mathbf{A}$). In the context of

multi-pumped TWPAs, these normalized metrics support the systematic verification of programmable cluster topologies and provide a robust benchmark for scalable measurement-based quantum information processing.

Another possible performance metric is the

- **Frobenius similarity:**

$$S_{\mathrm{F}}(\sigma, \Gamma) = \exp\left(-\frac{\|\sigma - \Gamma\|_{\mathrm{F}}^2}{\|\sigma\|_{\mathrm{F}}^2}\right), \tag{S47}$$

  where $\mathbf{\Gamma}$ and $\boldsymbol{\sigma}$ are both $2m \times 2m$ covariance matrices, which measures the similarity between the experimentally generated Gaussian state ($\mathbf{\Gamma}$) and the ideal target cluster state ($\boldsymbol{\sigma}$). The ideal covariance matrix $\boldsymbol{\sigma}$ can be constructed by applying the appropriate symplectic transformation associated with the adjacency matrix $\mathbf{A}$ to the vacuum state.

3.6 Losses

When modeling a lossy transmission line acting as a squeezer, the expression for the output covariance matrix requires refinement. In this case, the line can be described using an effective beam-splitter loss model [8], [9] and the Eq. S13 becomes

$$\hat{a}_{\mathrm{out},i}^{(\mathrm{loss})} = \sqrt{\eta_i} \sum_j \left(\mathbf{U}_{ij}\, \hat{a}_j + \mathbf{V}_{ij}\, \hat{a}_j^\dagger\right) + \sqrt{1-\eta_i}\, \hat{a}_i^{(\mathrm{th})}, \tag{S48}$$

where η_i denotes the transmission efficiency of the un-pumped JTWPA for mode i, and $\hat{a}_i^{(\mathrm{th})}$ represents the ladder operator associated with the thermal (noise) excitation injected through the second port of the beam splitter. This model leads to a modified output covariance matrix, related to that in Eq. S29 through

$$\boldsymbol{\sigma}_{\mathrm{loss}} = \mathbf{D}\, \boldsymbol{\sigma}\, \mathbf{D} + \left(\boldsymbol{I} - \mathbf{D}^2\right)\, \boldsymbol{\sigma}_{\mathrm{in}}, \tag{S49}$$

where

$$\mathbf{D} = \mathrm{diag}(\sqrt{\eta_1}, \ldots, \sqrt{\eta_N}, \sqrt{\eta_1}, \ldots, \sqrt{\eta_N})\,. \tag{S50}$$

As a consequence, Eq. S37 becomes

$$\mathbf{\Delta}_{\mathrm{loss}} = \mathbf{N}\mathbf{D}\, \boldsymbol{\sigma}\, \mathbf{D}\mathbf{N}^\top + \mathbf{N}(\mathbf{I} - \mathbf{D}^2)\boldsymbol{\sigma}_{\mathrm{in}}\mathbf{N}^\top\,, \tag{S51}$$

When the nullifier variances (the diagonal elements of $\mathbf{\Delta}$) are initially squeezed, losses reduce the degree of squeezing. For simplicity, we consider the case of uniform transmission efficiency, $\mathbf{D} = \eta\, \mathbf{I}_{2N}$, so that

$$\begin{aligned}\mathbf{\Delta}_{\text{loss}} - \mathbf{\Delta} &= \big(\eta\mathbf{\Delta} + (1-\eta)\,\mathbf{N}\boldsymbol{\sigma}_{\text{in}}\mathbf{N}^{\top}\big) - \mathbf{\Delta} &\text{(S52)}\\ &= (\eta - 1)\,\mathbf{\Delta} + (1-\eta)\,\mathbf{N}\boldsymbol{\sigma}_{\text{in}}\mathbf{N}^{\top} &\text{(S53)}\\ &= (1-\eta)\big(\mathbf{N}\boldsymbol{\sigma}_{\text{in}}\mathbf{N}^{\top} - \mathbf{\Delta}\big)\,. &\text{(S54)}\end{aligned}$$

Since for a thermal input state $(\mathbf{N}\boldsymbol{\sigma}_{\text{in}}\mathbf{N}^{\top})_{ii} \geq (\mathbf{\Delta}_{\text{vac}})_{ii}$, and the nullifier variances are initially squeezed ($\mathbf{\Delta}_{ii} \leq (\mathbf{\Delta}_{\text{vac}})_{ii}$), it follows that $(\mathbf{\Delta}_{\text{loss}})_{ii} > \mathbf{\Delta}_{ii}$ for all i, i.e., losses increase the nullifier variances and therefore reduce the squeezing.

4 Reference State Protocol and Covariance Matrix Reconstruction

In typical continuous-variable experiments, the measurement of quadratures via homodyne or heterodyne detection requires a well-defined phase reference, set by the LO. Similarly, the generation of squeezed states via parametric processes depends on the phase of the pump field, which defines the squeezing angle. In the absence of a stable or known phase relation between the pump and the LO, the measured quadratures are rotated by unknown phases θ_i, and the observed statistics correspond to an ensemble over these unknown local phases.

However, it is still possible to reconstruct the covariance matrix of the output Gaussian state $\mathbf{\Gamma}$ by exploiting the structure of quantum correlations. This approach provides a data-driven method to self-calibrate the phase reference.

4.1 Scaled Covariance Matrix

In the notation defined in Eq. S20, the quadrature operators after the amplification chain of the j-th mode $\hat{I}_j$ and $\hat{Q}_j$ can be modeled as:

$$\hat{I}_j + i\hat{Q}_j = \sqrt{2}\left(\sqrt{G_{\text{SYS},j}}\hat{a}_j + \sqrt{G_{\text{SYS},j} - 1}\,\hat{h}_j\right) \simeq \sqrt{2\,G_{\text{SYS},j}}\,(\hat{a}_j + \hat{h}_j), \quad G_{\text{SYS},j} \gg 1. \tag{S55}$$

where $G_{\text{SYS},j}$ denotes the system gain for the j-th spectral mode, $\hat{a}_j$ describes the j-th mode at the JTWPA output and $\hat{h}_j$ is an effective bosonic operator describing the noise added by the amplification chain.

The raw voltage data ($I_j^{(\text{raw})}$, $Q_j^{(\text{raw})}$) acquired from the measurement system are scaled to quadrature operators expressed in photon-units (i_j, q_j) via the relations:

$$i_j = \frac{I_j^{(\text{raw})}}{\sqrt{G_{\text{SYS},j} Z_0 \hbar\omega_j \tau^{-1}/2}}, \quad q_j = \frac{Q_j^{(\text{raw})}}{\sqrt{G_{\text{SYS},j} Z_0 \hbar\omega_j \tau^{-1}/2}} \tag{S56}$$

where $Z_0 = 50\ \Omega$ is the characteristic impedance of the transmission line, $\omega_j/2\pi$ is the mode frequency, and $\tau = 6\ \mu$s is the acquisition time.

From repeated measurements, we construct the measured scaled covariance matrix elements:

$$\Gamma^{\text{exp}}_{j,l} = \frac{1}{2}\langle M_j M_l + M_l M_j\rangle - \langle M_j\rangle\langle M_l\rangle\,. \tag{S57}$$

where $\boldsymbol{M} = (i_1, q_1, \ldots, i_m, q_m)^T$. This construction of the M vector is in a mode-wise ordering. The covariance matrix in the yet unknown θ-rotated basis $\mathbf{\Gamma}_{\boldsymbol{\theta}}$ of the two-mode quantum state generated at the output of the JTWPA is reconstructed following a reference state subtraction method [10] [11]:

$$\mathbf{\Gamma}_{\boldsymbol{\theta}} = (\mathbf{\Gamma}^{\text{exp}}_{\mathbf{ON}} - \mathbf{\Gamma}^{\text{exp}}_{\mathbf{OFF}}) + \boldsymbol{\sigma}^{\text{in}}, \tag{S58}$$

where $\mathbf{\Gamma}^{\text{exp}}_{\mathbf{ON}}$ and $\mathbf{\Gamma}^{\text{exp}}_{\mathbf{OFF}}$ are the scaled covariance matrices measured with the pumps on and off, respectively, and $\boldsymbol{\sigma}_{\text{in}} = \frac{1}{2}\mathbb{I}\coth\frac{\hbar\omega_j}{2k_BT}$ is the covariance matrix of the input thermal state, which reduces to the vacuum state defined in Eq. S24 in the limit $k_BT \ll \hbar\omega_j$.

The reference-state subtraction is a standard method to cancel out the noise added by the amplification chain, assuming the mode described by the operator $\hat{h}_j$ is in a thermal state [10] [11]. This assumption is confirmed by the off-diagonal elements of $\mathbf{\Gamma}^{\text{exp}}_{\mathbf{OFF}}$, all of which have Pearson coefficients below 0.005, consistent with a diagonal thermal covariance matrix.

Since the choice on the LO phases is arbitrary, the reconstructed covariance matrix $\mathbf{\Gamma}_{\boldsymbol{\theta}}$ is in a yet undefined θ-rotated basis $\boldsymbol{X}^{(\text{exp})}_{\boldsymbol{\theta}}$ such that

$$\boldsymbol{X}^{(\text{exp})}_{\boldsymbol{\theta}} = \mathbf{R}_{\boldsymbol{\theta}} \cdot \boldsymbol{X}^{(\text{exp})}, \qquad \mathbf{\Gamma}_{\boldsymbol{\theta}} = \mathbf{R}_{\boldsymbol{\theta}} \cdot \mathbf{\Gamma} \cdot \mathbf{R}^{\text{T}}_{\boldsymbol{\theta}}, \qquad \boldsymbol{R}_{\boldsymbol{\theta}} = \bigoplus_{j=1}^{m} \begin{pmatrix} \cos\theta_j & -\sin\theta_j \\ \sin\theta_j & \cos\theta_j \end{pmatrix}. \tag{S59}$$

where $\boldsymbol{X}^{\text{exp}}$ is the experimental cluster state basis expressed in the mode-wise notation. However, by numerically inverting Eq. S59, we can reconstruct the output covariance matrix.

4.2 System Gain Calibration

The system gain G_j and system noise temperature $T_{\text{SYS,j}}$ for each spectral mode are calibrated by measuring the noise power spectral density at the output of a variable temperature stage (VTS) as a function of its temperature T_{VTS}.

The measured noise power is fitted to the following expression where G_j and $T_{\text{SYS,j}}$ are the unknown parameters:

$$\frac{\langle I_j^2 + Q_j^2 \rangle}{Z_0 \tau_j^{-1}} = G_{\text{SYS},j} \left[\frac{\hbar\omega_j}{2} \coth\left(\frac{\hbar\omega_j}{2k_{\text{B}}T_{\text{VTS}}} \right) + k_{\text{B}}T_{\text{SYS,j}} \right]. \tag{S60}$$

Here I_j and Q_j are the in-phase and quadrature voltage components, respectively, ω_j is the mode frequency, and k_{B} is the Boltzmann constant.

4.3 Uncertainty budget

In Sec. 4.1 we described the reconstruction of the output covariance matrix $\mathbf{\Gamma}$ from the experimental data. To estimate the uncertainties on the derived quantities—such as the

two-mode squeezing, the nullifiers, and the optimized gains—we propagated the dominant experimental contributions entering the reconstruction formulas.

Specifically, we considered: (i) statistical (Type A) fluctuations of the measured covariance matrices Γ^{exp}, defined in Eq. S57; (ii) the uncertainty on the system gain G_{sys} entering the quadrature normalization in Eq. S56 (obtained from Planck spectroscopy fits, see Sec. 4.2); and (iii) the uncertainty on the cryogenic temperature appearing in Eq. S58.

The relatively large gain uncertainty (about 15 %) and the non-linear dependence of the squeezing and nullifier functions on G_{sys} make analytical propagation of errors impractical, justifying the Monte Carlo approach. Statistical errors were estimated from $N = 5 \times 10^6$ repetitions of each experiment, assuming independence between ON and OFF measurements and Gaussian-distributed data. Gain and temperature uncertainties were sampled according to Gaussian and uniform distributions, respectively.

Supplementary Table 1: Experimental uncertainty budget

Quantity	Value	Standard uncertainty
Gain G_{sys}	59.1 dB	0.6 dB
Cryostat temperature T_{cr}	70 mK	10 mK / $\sqrt{3}$
Covariance matrices $\Gamma_{ij}^{\mathrm{exp,ON}}$, $\Gamma_{ij}^{\mathrm{exp,OFF}}$	–	estimated from $N = 5 \times 10^6$ samples

4.4 JTWPA gain estimation

In our multi-pump configuration, the amplification of mode i arises from the collective contribution of concurrent nonlinear processes. We define an operational effective gain G_j that treats the system as a generalized phase-insensitive amplifier. This approach, grounded in the framework established by Caves [12], allows us to account for the total variance enhancement while intrinsically incorporating the added noise (G_j-1) necessitated by the preservation of the bosonic commutation relations:

$$G_j = \frac{1}{2}\left(\frac{\Gamma_{\theta,jj}}{\sigma_{jj}^{\mathrm{in}}} + 1\right). \tag{S61}$$

By referencing the measured output covariance $\Gamma_{\theta,ii}$ to the calibrated input variance $\sigma_{ii}^{\mathrm{in}}$, this formulation ensures that the extracted gain remains robust against the quantum-limited noise floor inherent to the amplification process. In Supplementary Figure 5, the average gain of the multi-pumped JTWPA, is plotted as a function of the single pump powers for four different topologies.

4.5 Gaussianity Validation

To validate that the measured quantum states retain Gaussian character—a critical assumption underpinning cluster-state quantum computation with continuous variables—we

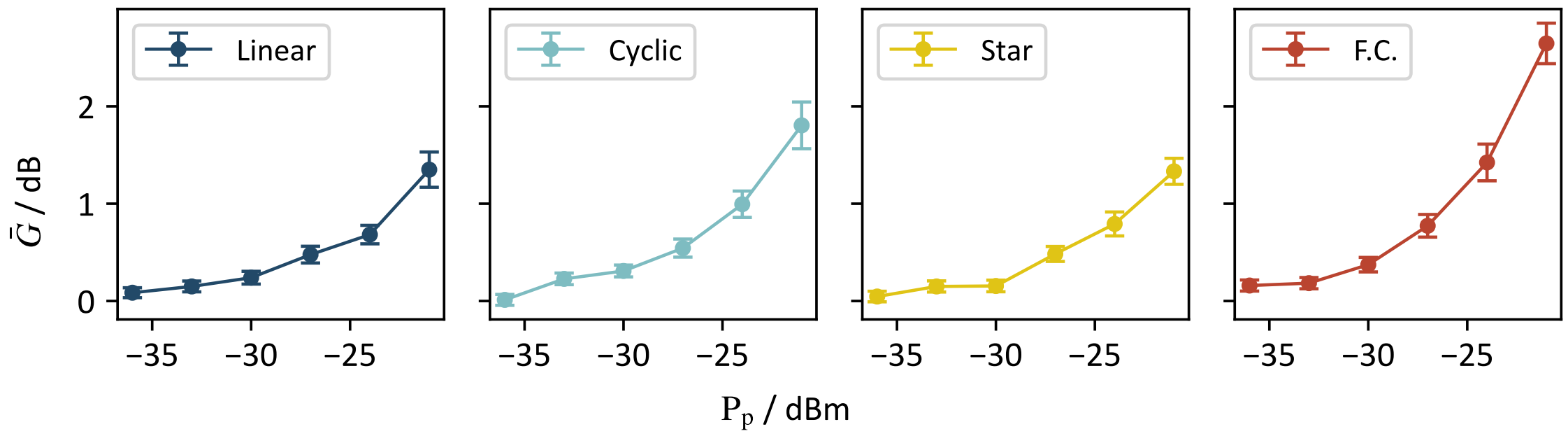

Supplementary Figure 5: **JTWPA gain in multi-pumped regime.** Gain of the JTWPA G_i, averaged over frequencies ω_i, as a function of the single pump power for different cluster topologies. The error bars show the estimated standard deviation.

performed a comprehensive statistical analysis on a representative subset of quadrature data collected via heterodyne detection. The analysis is here reported focusing, as an example, on the cyclic four-mode cluster configuration presented in Supplementary Figure 3(b,f) of the main text and includes:

- **Inspection of marginal histograms** to verify Gaussian-shaped distributions (see Supplementary Figure 6).
- **Moment analysis up to fourth order**, including skewness, excess kurtosis, and fourth-order cumulants (see Supplementary Figure 7).
- **Standard normality tests**, namely the Shapiro–Wilk and Anderson–Darling tests (see Supplementary Figure 8).

All results confirm that the measured quadrature distributions are statistically indistinguishable from ideal Gaussians within experimental uncertainty. This supports the validity of Gaussian-state formalism and covariance-based methods used throughout the entanglement and nullifier analysis.

4.6 Multiplexed Heterodyne Detection

The calibration and validation procedures were performed using a broadband heterodyne detection system with simultaneous acquisition of multiple frequency modes. LOs were carefully placed to avoid image band contamination and were phase-locked to ensure consistent quadrature references across the entire spectral span.

This multiplexed detection strategy is crucial to resolve all entangled modes in a single shot, enabling full reconstruction of the multimode covariance matrix with minimal overhead.

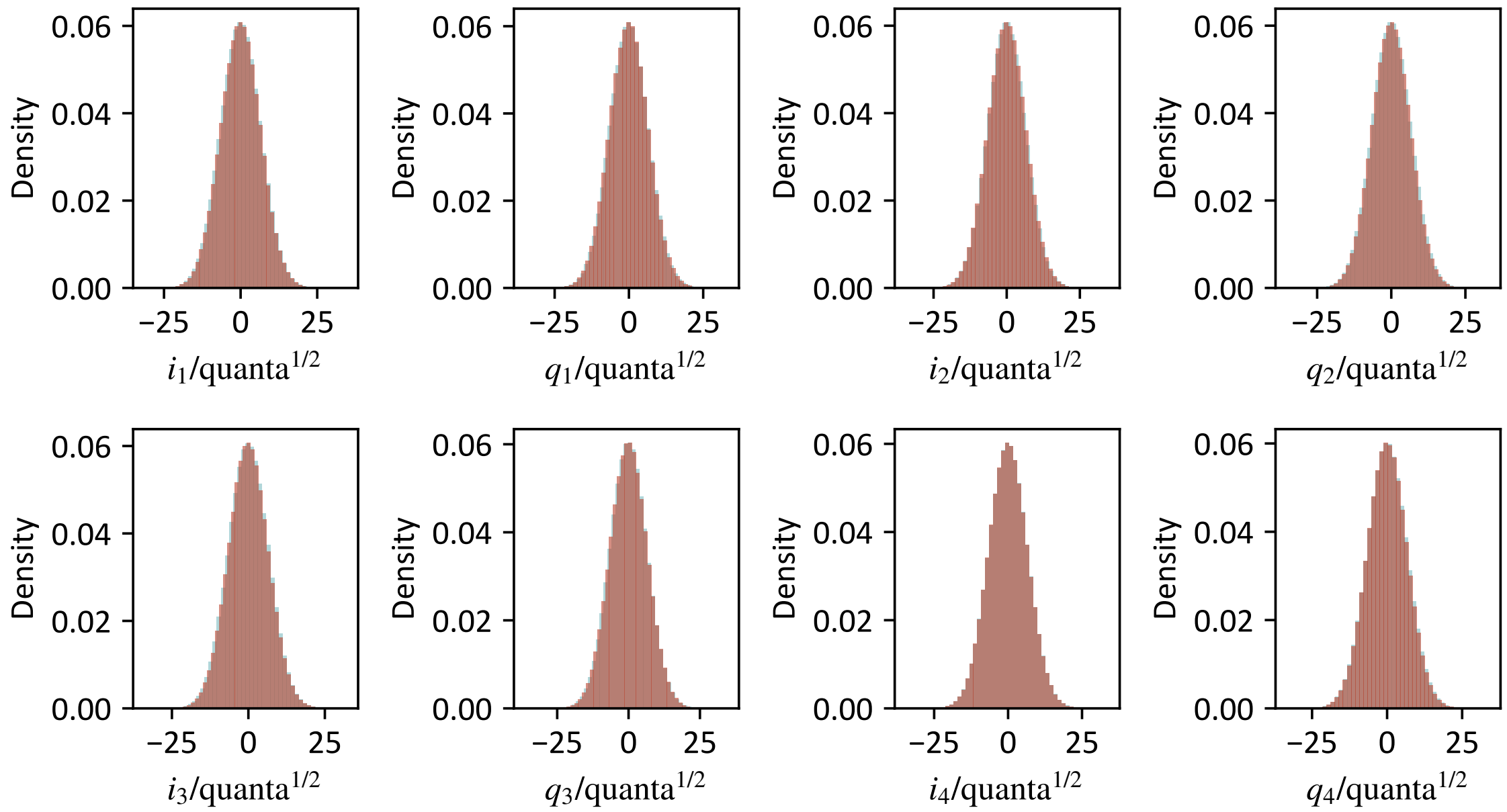

Supplementary Figure 6: **Gaussian quadrature distributions of the four measured modes in the linear cluster state.** Heterodyne detection of the four modes yields Gaussian distributions for each quadrature, consistent with the expected statistics of squeezed states forming the linear cluster. The data correspond to the same cluster configuration shown in Supplementary Figure 3(b,f) of the main text. The measured variances are used to reconstruct the covariance matrix and assess entanglement through the nullifier variances.

4.7 Optimization Strategy: Maximizing Inter-mode Covariances

Our optimization strategy consists in the maximization of $p_j - x_l$ correlations over the set of unknown local oscillator phases $\boldsymbol{\theta}$, effectively determining the optimal global quadrature basis in which the reconstructed covariance matrix $\boldsymbol{\Gamma}_\theta$ most closely resembles an ideal cluster state. To this end, we maximize

$$\mathcal{C}_{px}(\theta) = \frac{1}{m^2} \sum_{j=1}^{m} \sum_{l=1}^{m} \boldsymbol{\Gamma}_{\theta,p_j,x_l} \tag{S62}$$

which is the mean value of all pairwise covariances between the p_j and x_l quadratures in the rotated covariance matrix $\boldsymbol{\Gamma}_\theta$.

Crucially, the set of phases $\boldsymbol{\theta}$ of the LOs obtained by this optimization are not arbitrary: they are directly related to the unknown pump phases used to generate squeezing in the source. These pump phases determine the orientation of the squeezing ellipse in

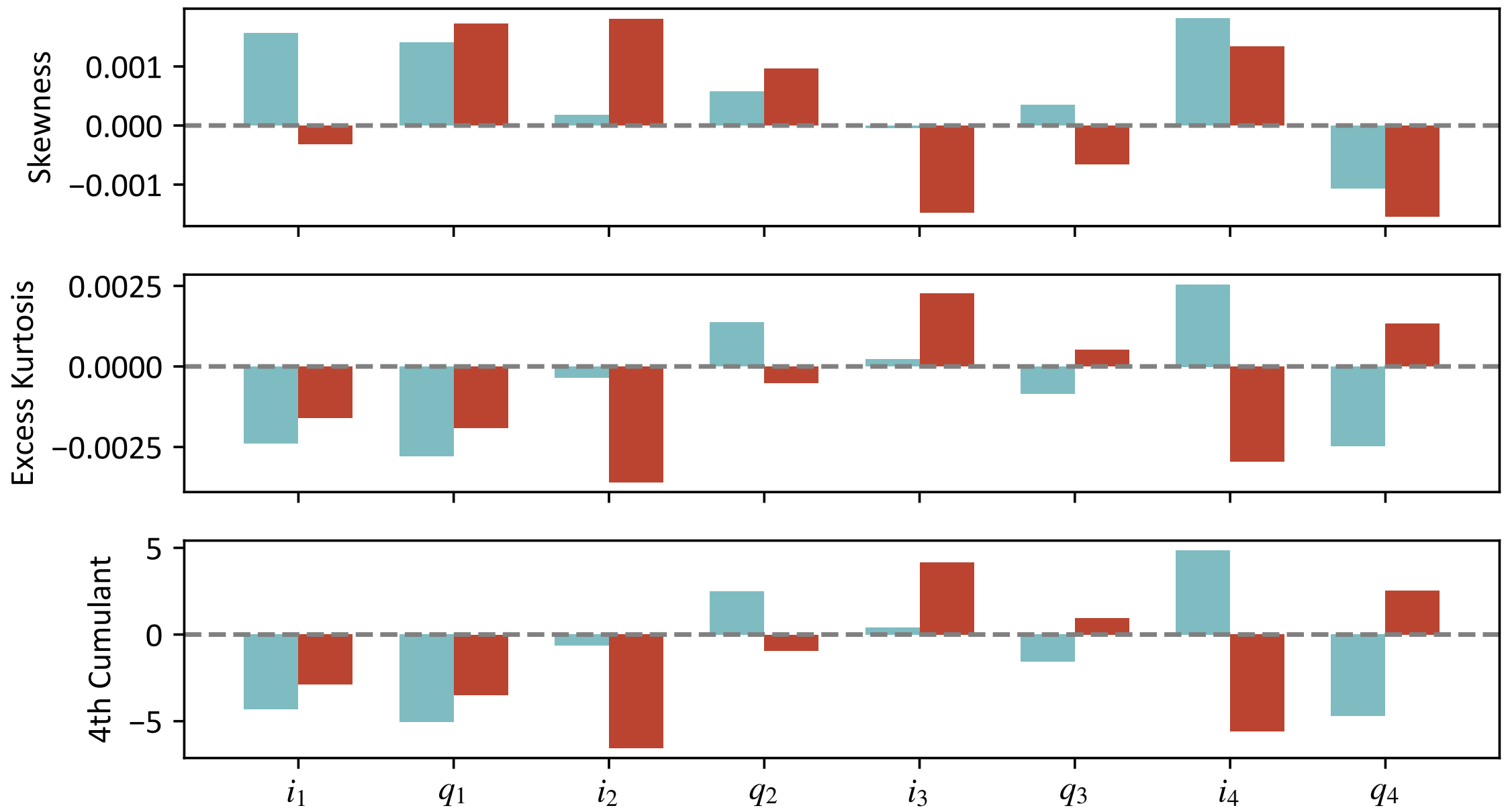

Supplementary Figure 7: **Higher-order statistical moments of the measured quadratures for the linear cluster state.** We report the skewness, excess kurtosis, and fourth-order cumulant of the quadrature distributions for the four measured modes, comparing the vacuum reference state (pump off, light blue) and the cluster state (pump on, red), as shown in Supplementary Figure 3(b,f) of the main text. In all cases, the skewness remains below 0.002 and the excess kurtosis below 0.0025, confirming the near-Gaussian character of the distributions and validating the Gaussian cluster state assumption underlying the nullifier-based analysis.

phase space, i.e., the squeezing angle, and are typically not accessible through direct measurement. As a result, the effective squeezing observed via heterodyne detection depends on the alignment between the LO phase and the unknown squeezing angle. Optimizing $\boldsymbol{\theta}$ thus serves not only to reveal the strongest squeezing present in the system, but also to compensate for unknown phase shifts introduced during state preparation.

4.8 Reconstruction of the Covariance Matrix and Comparison with Theoretical Model

Once the optimal angle

$$\boldsymbol{\theta}_{\text{opt}} = \arg\max_{\theta} \mathcal{C}_{px}(\theta)$$

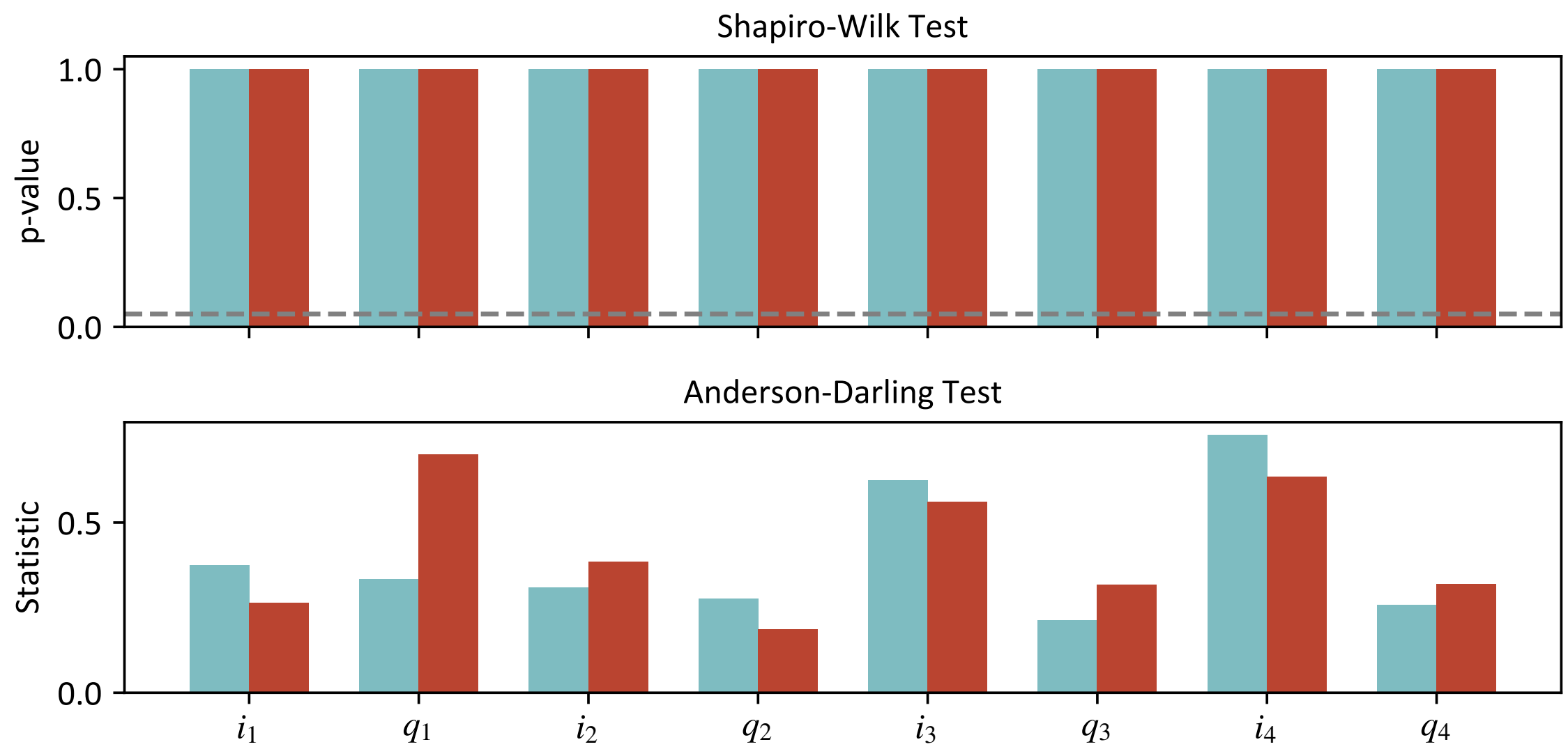

Supplementary Figure 8: **Normality tests on quadrature distributions for the linear cluster state.** We show the results of two standard normality tests—Shapiro–Wilk and Anderson–Darling—applied to the quadrature distributions of the four measured modes, for both the vacuum reference state (pump off, light blue) and the cluster state (pump on, red), corresponding to the configuration analyzed in Supplementary Figure 3(b,f) in the main text. The Shapiro–Wilk test yields p-values close to 1 for all modes, indicating no statistically significant deviation from normality. The Anderson–Darling test statistics remain between 0.25 and 0.5 in all cases, further supporting the Gaussian character of the measured states and justifying the use of covariance-based criteria in the entanglement analysis.

is found, the covariance matrix in the original basis can be estimated by applying the inverse of the rotation matrix:

$$\mathbf{\Gamma} = \mathbf{R}_{\boldsymbol{\theta}_{\mathrm{opt}}}^{-1} \mathbf{\Gamma}_{\theta} \left(\mathbf{R}_{\boldsymbol{\theta}_{\mathrm{opt}}}^{-1} \right)^{\top}, \tag{S63}$$

This procedure, for which we report the effects in Supplementary Figure 9, effectively recovers a global phase reference, aligning both the measured quadratures and the squeezing direction. It is valid provided that the output state exhibits sufficient two-mode correlations.

We then compared the reconstructed covariance matrix Γ with its theoretical counterpart σ using the Frobenius similarity introduced in Eq. S47. The theoretical covariance matrices were computed using the formalism presented in Section 3.4, including the effects of JTWPA losses as described in Section 3.6. The measured total loss of the device and

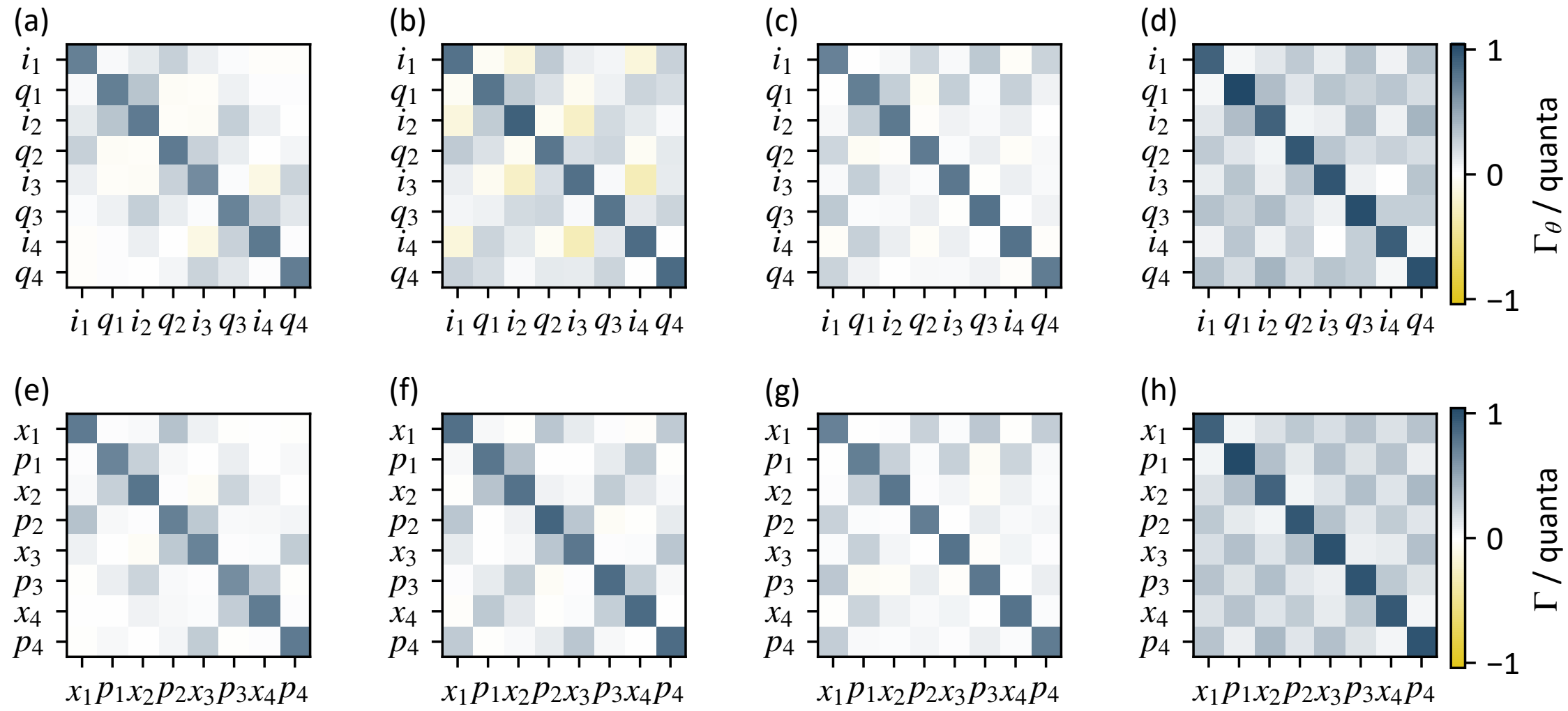

Supplementary Figure 9: **Effect of the optimization strategy based on $p - x$ correlation maximization of $\boldsymbol{\Gamma_\theta}$.** Covariance matrices reported in Supplementary Figure 3 of the paper prior (a-d) and post (e-h) the optimization algorithm reported in Subsection 4.8.

its packaging, including connections to electromechanical switches, is $\eta = -1$ dB. The comparison is shown in Supplementary Figure 10.

4.9 Discussion

The reconstruction protocol presented here addresses the challenge of unknown quadrature rotations arising from phase delays accumulated during signal propagation through the readout chain. While the local oscillators and the pump tones are phase-locked to a common reference—ensuring overall coherence—the detection phases at different frequencies can experience distinct, frequency-dependent shifts due to dispersive and thermal effects in the measurement path. These shifts are typically unknown and difficult to calibrate directly.

By exploiting inter-mode quantum correlations, in particular those induced by two-mode squeezing, we implement a data-driven optimization strategy that compensates for these residual phase offsets. The optimal set of local quadrature angles is determined numerically by maximizing the off-diagonal correlations in the rotated covariance matrix $\boldsymbol{\Gamma}_\theta$. This allows us to reconstruct the physical covariance matrix $\boldsymbol{\Gamma}$ in a consistent global quadrature basis.

The validity of this reconstruction is confirmed by several independent checks: the observed quadrature statistics remain indistinguishable from ideal Gaussian distributions, and the reconstructed covariance matrices match closely with theoretical predictions. This

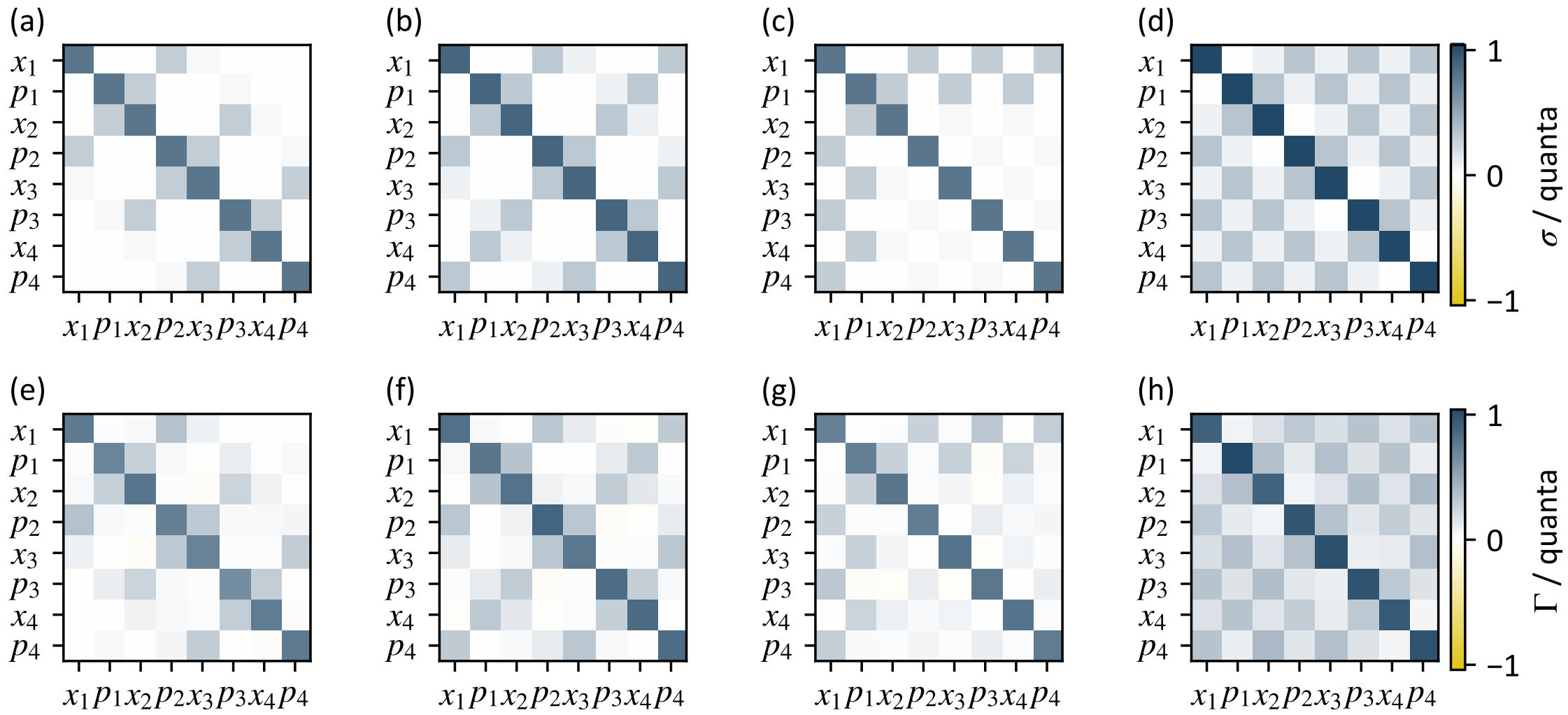

Supplementary Figure 10: **Comparison between simulated and experimental covariance matrices.** Theoretical covariance matrices (panels a–d) correspond to simulations (see the Appendix) performed using the squeezing parameter r that maximizes the Frobenius similarity defined in Eq. S47. Experimental covariance matrices reconstructed from data are shown in panels (e–h). The optimized squeezing parameters used in the simulations are, respectively, $|r| = 0.275, 0.283, 0.278$, and 0.289 for panels (a) through (d). The maximum relative deviation of $|r|$ is 5%, confirming the robustness and consistency of the parameter estimation across different experiments.

ensures that key properties of the state, such as entanglement structure and nullifier variances, can be reliably extracted.

Importantly, this protocol requires no additional calibration hardware and is compatible with broadband, multiplexed detection schemes. It is therefore particularly suited to integrated or cryogenic platforms, where frequency-dependent phase delays are common and in-situ phase calibration is challenging. The method provides a robust and scalable approach to covariance matrix reconstruction for high-dimensional Gaussian states in realistic experimental settings.

A Examples of Covariance Matrices Analytical Calculations

In practical implementations of multimode Gaussian cluster states using multi-pump traveling wave parametric amplifiers (JTWPAs), it is common for *ancillary modes*—modes not explicitly part of the target cluster graph—to become entangled with the main cluster modes via two-mode squeezing (TMS) processes. These ancillary couplings can significantly affect the output covariance matrix by modifying modal gain distributions and introducing residual correlations (see Supplementary Figure 11).

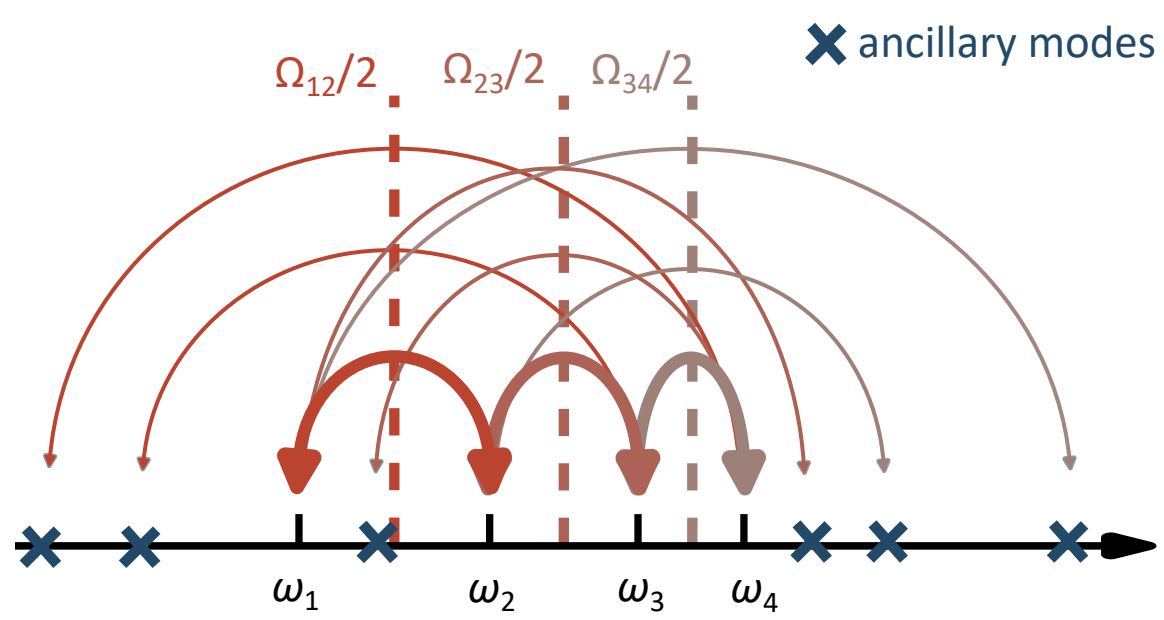

Supplementary Figure 11: **Ancillary Modes in a Linear Cluster State.** Example of ancillary modes in a linear cluster state. Although these modes are not part of the intended cluster topology, they do participate in the amplification process due to their coupling with connected modes via the pump tones.

For accurate fidelity estimation and nullifier optimization, it is therefore essential to include these ancillary modes in the theoretical modeling.

Coherently with the proposed theoretical framework, all simulations were carried out in block-wise notation and subsequently converted into the mode-wise notation by applying the transformation

$$\boldsymbol{\sigma}_{\text{mode}} = \mathbf{P}\,\boldsymbol{\sigma}_{\text{block}}\,\mathbf{P}^{\top}, \tag{S64}$$

where

$$\mathbf{P} = \sum_{j=1}^{m} \left(\mathbf{e}_{2j-1}\mathbf{e}_j^{\top} + \mathbf{e}_{2j}\mathbf{e}_{m+j}^{\top} \right), \tag{S65}$$

and $\mathbf{e}_k$ denotes the standard basis column vector of dimension $2m$, with a 1 in position k and 0 elsewhere.

A.1 Quadrature Rotation

In our simulations of two-mode squeezing generated by a JTWPA, the strongest correlations emerge along the x–x quadratures of the two modes. To express the covariance matrices in a cluster-type basis, we apply a local rotation of $\theta = \pi/4$ to each mode. This transformation

maps the maximal x–x correlations into standard p–x cluster-type correlations, facilitating comparison with ideal Gaussian cluster states.

This corresponds to a symplectic transformation on the output covariance matrix $\boldsymbol{\sigma}$:

$$\boldsymbol{\sigma}_{\boldsymbol{\theta}} = \mathbf{R}_{\boldsymbol{\theta}} \cdot \boldsymbol{\sigma} \cdot \mathbf{R}_{\boldsymbol{\theta}}^{\mathrm{T}} \tag{S66}$$

with $\mathbf{R}_\theta$ defined in Eq. S59. All explicit covariance matrices reported below are expressed in this rotated (mode-wise) basis.

A.2 Linear Four-Mode Cluster with Distributed Ancillary Coupling: Covariance Matrix

We now present the symbolic expression for the reduced covariance matrix of a linear four-mode cluster state with six ancillary couplings. This result is obtained by appropriately choosing the adjacency matrix $\mathbf{A}$ and following the procedure described in Eqs. S32 and S34.

This configuration involves ten modes in total:

- Principal modes: $1-4$, connected linearly via TMS links $(1,2),(2,3),(3,4)$.
- Ancillary modes: $5-10$, connected to the principal chain via TMS links:

$$(1,5),\ (1,6),\ (2,7),\ (3,8),\ (4,9),\ (4,10).$$

Assuming a uniform squeezing parameter r, we define the following symbolic quantities:

$$\begin{aligned}
D &= -6\cosh(|r|) + 3\cosh(2|r|) + \frac{7}{2},\\
A &= (3\cosh(|r|) - 2)\sinh(|r|),\\
E &= \tfrac{1}{2}\sinh^2(|r|).
\end{aligned}$$

The reduced covariance matrix for the cluster modes $1-4$, in the rotated mode-wise basis, is:

$$\boldsymbol{\sigma}_{\mathrm{red}}^{(1:4)} = \begin{bmatrix}
D & 0 & 0 & A & E & 0 & 0 & 0\\
0 & D & A & 0 & 0 & E & 0 & 0\\
0 & A & D & 0 & 0 & A & E & 0\\
A & 0 & 0 & D & A & 0 & 0 & E\\
E & 0 & 0 & A & D & 0 & 0 & A\\
0 & E & A & 0 & 0 & D & A & 0\\
0 & 0 & E & 0 & 0 & A & D & 0\\
0 & 0 & 0 & E & A & 0 & 0 & D
\end{bmatrix}$$

A.3 Cyclic Four-Mode Cluster State with Ancillary Modes: Covariance Matrix

We now extend the theoretical model of a cyclic four-mode cluster state to include ancillary modes entangled via two-mode squeezing (TMS). This configuration reflects realistic conditions in multi-pump JTWPA platforms, where each principal mode is coupled to additional spectral modes through engineered or parasitic squeezing.

We consider a system of twelve modes:

- Principal modes: $1-4$, forming a closed ring via TMS links $(1,2),(2,3),(3,4),(4,1)$.
- Ancillary modes: $5-12$, coupled to the principal modes as follows:

$$(1,5),(1,6), \quad (2,7),(2,8), \quad (3,9),(3,10), \quad (4,11),(4,12)$$

Assuming uniform squeezing parameter r, we define the following symbolic coefficients:

$$\begin{aligned} D &= -12\cosh(|r|) + 5\cosh(2|r|) + \frac{15}{2}, \\ A &= (4\cosh(|r|) - 3)\sinh(|r|), \\ E &= \sinh^2(|r|). \end{aligned}$$

Then the reduced covariance matrix for modes 1–4, in the mode-wise rotated quadrature basis, is:

$$\boldsymbol{\sigma}_{\text{red}}^{(1:4)} = \begin{bmatrix} D & 0 & 0 & A & E & 0 & 0 & A \\ 0 & D & A & 0 & 0 & E & A & 0 \\ 0 & A & D & 0 & 0 & A & E & 0 \\ A & 0 & 0 & D & A & 0 & 0 & E \\ E & 0 & 0 & A & D & 0 & 0 & A \\ 0 & E & A & 0 & 0 & D & A & 0 \\ 0 & A & E & 0 & 0 & A & D & 0 \\ A & 0 & 0 & E & A & 0 & 0 & D \end{bmatrix}$$

A.4 Star Four-Mode Cluster State with Ancillary Modes: Covariance Matrix

We now consider a star-shaped cluster configuration extended to include ancillary modes coupled via two-mode squeezing (TMS) to the outer (leaf) modes. This scenario models a realistic system where the central hub remains clean while each peripheral mode connects to external spectral modes.

The system comprises ten modes in total:

- Principal modes: $1-4$, arranged such that mode 1 is connected to $2,3,4$.

- Ancillary modes: 5 − 10, with TMS couplings:

$$(2,5),(2,6), \quad (3,7),(3,8), \quad (4,9),(4,10)$$

Assuming a uniform squeezing parameter r, we define:

$$\begin{aligned} D &= -6\cosh(|r|) + 3\cosh(2|r|) + \frac{7}{2}, \\ A &= (3\cosh(|r|) - 2)\sinh(|r|), \\ E &= \tfrac{1}{2}\sinh^2(|r|). \end{aligned}$$

Then, the reduced covariance matrix for the principal cluster modes 1–4, in the rotated mode-wise quadrature basis, is:

$$\boldsymbol{\sigma}_{\text{red}}^{(1:4)} = \begin{bmatrix} D & 0 & 0 & A & 0 & A & 0 & A \\ 0 & D & A & 0 & A & 0 & A & 0 \\ 0 & A & D & 0 & E & 0 & E & 0 \\ A & 0 & 0 & D & 0 & E & 0 & E \\ 0 & A & E & 0 & D & 0 & E & 0 \\ A & 0 & 0 & E & 0 & D & 0 & E \\ 0 & A & E & 0 & E & 0 & D & 0 \\ A & 0 & 0 & E & 0 & E & 0 & D \end{bmatrix}$$

A.5 Fully Connected Four-Mode Cluster State with Ancillary Modes: Covariance Matrix

We now consider a fully connected four-mode cluster state where each principal mode is coupled via two-mode squeezing (TMS) to three ancillary modes. This configuration models heavy parasitic interactions common in over-pumped JTWPA systems.

We consider a total of sixteen modes:

- Principal modes: 1 − 4, fully connected among themselves via TMS links.
- Ancillary modes: 5 − 16, with each cluster mode coupled to three ancillary modes as follows:

$$\text{Mode 1: } (5,6,7), \quad \text{Mode 2: } (8,9,10), \quad \text{Mode 3: } (11,12,13), \quad \text{Mode 4: } (14,15,16)$$

Assuming uniform squeezing strength r, we define the following symbolic expressions:

$$\begin{aligned} D &= -30\cosh(|r|) + \frac{21}{2}\cosh(2|r|) + 20, \\ A &= (6\cosh(|r|) - 5)\sinh(|r|), \\ E &= \sinh^2(|r|). \end{aligned}$$

Then the reduced covariance matrix for the principal modes 1–4, in mode-wise (rotated) block-wise order, is:

$$\boldsymbol{\sigma}_{\text{red}}^{(1:4)} = \begin{bmatrix} D & 0 & E & A & E & A & E & A \\ 0 & D & A & E & A & E & A & E \\ E & A & D & 0 & E & A & E & A \\ A & E & 0 & D & A & E & A & E \\ E & A & E & A & D & 0 & E & A \\ A & E & A & E & 0 & D & A & E \\ E & A & E & A & E & A & D & 0 \\ A & E & A & E & A & E & 0 & D \end{bmatrix}$$

Supplementary References

[1] Ferrero, A. & Pisani, U. Two-port network analyzer calibration using an unknown 'thru'. *IEEE Microwave and Guided Wave Letters* **2**, 505–507 (1992).

[2] Oberto, L. *et al.* Measurement and calibration approaches for full two-port scattering parameters at mk temperatures (2025). `2505.19922`.

[3] Alocco, A. *et al.* Towards Quantifying Two-Mode Correlation Linewidths in Quantum Circuits. In *2025 International Workshop on Integrated Nonlinear Microwave and Millimetre-Wave Circuits (INMMIC)*, 1–4 (2025).

[4] Perelshtein, M. *et al.* Broadband Continuous-Variable Entanglement Generation Using a Kerr-Free Josephson Metamaterial. *Phys. Rev. Appl.* **18**, 024063 (2022).

[5] Frattini, N. E. *et al.* 3-wave mixing Josephson dipole element. *Applied Physics Letters* **110**, 222603 (2017).

[6] Menicucci, N. C. *et al.* Universal quantum computation with continuous-variable cluster states. *Phys. Rev. Lett.* **97**, 110501 (2006).

[7] Alexander, R. N. *et al.* One-way quantum computing with arbitrarily large time-frequency continuous-variable cluster states from a single optical parametric oscillator. *Phys. Rev. A* **94**, 032327 (2016).

[8] Houde, M., Govia, L. & Clerk, A. Loss Asymmetries in Quantum Traveling-Wave Parametric Amplifiers. *Phys. Rev. Appl.* **12**, 034054 (2019).

[9] Flurin, E., Roch, N., Mallet, F., Devoret, M. H. & Huard, B. Generating Entangled Microwave Radiation Over Two Transmission Lines. *Phys. Rev. Lett.* **109**, 183901 (2012).

[10] Eichler, C. *et al.* Observation of two-mode squeezing in the microwave frequency domain. *Phys. Rev. Lett.* **107**, 113601 (2011).

[11] Flurin, E., Roch, N., Pillet, J. D., Mallet, F. & Huard, B. Superconducting Quantum Node for Entanglement and Storage of Microwave Radiation. *Phys. Rev. Lett.* **114**, 090503 (2015).

[12] Caves, C. M. Quantum limits on noise in linear amplifiers. *Phys. Rev. D* **26**, 1817–1839 (1982).
