# Supplementary material for: Programmable microwave cluster states via Josephson metamaterials
Source: Nat Commun. 2026 Jun 18;17:7700. doi: 10.1038/s41467-026-74377-2 (PMC13434799; doi:10.1038/s41467-026-74377-2)
Supplement: Supplementary file 2 [file 41467_2026_74377_MOESM2_ESM.pdf]

nature portfolio

Peer Review File

Programmable Microwave Cluster States via Josephson Metamaterials

Corresponding Author: Dr Emanuele Enrico

This file contains all reviewer reports in order by version, followed by all author rebuttals in order by version.

Version 0:

Reviewer comments:

Reviewer #1

(Remarks to the Author)
In their paper “Programmable Microwave Cluster States via Josephson Metamaterials” the authors experimentally demonstrate the generation of several Continuous Variable (CV) cluster states in the microwave domain. Such states are essential for quantum information processing with continuous variables. They can enable quantum computation that is based on Gaussian states of electromagnetic radiation, deterministic operations, and high-fidelity measurements. To generate these states, the authors use a Josephson metamaterial in the form of a Josephson Traveling wave parametric amplifier (JTWPA). They operate the device in three-wave mixing mode, and apply multiple pump tones to the device simultaneously, which based on their frequencies generate two-mode squeezing states between pairs of nondegenerate modes within the JTWPA bandwidth. By varying the number of applied pump tones and engineering their frequencies, the authors are able to “program” the JTWPA to produce CV cluster states that realize different connectivity configurations between the entangled modes.

In general, the results of the paper are important and interesting to a broad audience. The paper and supplementary information are clear and well written. The measurements are carefully executed and adequately analyzed. I think the paper is a good candidate for publication in Nature Communications after addressing several important issues in a revised version.

Main issues:
1- In their paper, the authors do not provide sufficient perspective or assessment regarding the results they achieve. Although they present several performance metrics such as the nullifier measurement and Frobenius distance, they do not put these values in context for non-expert readers to understand whether they are good or not. For example, can you compare these values to the ones measured in the optical domain? Are these values sufficient for performing scalable or even basic CV quantum computation in the microwave domain? How far off are they?
2- In their paper, the authors cite several limitations of their device and setup and mention several ways to overcome them but do not provide any quantitative estimates of these limitations or explain how they are affecting the measured performance and to what extent. They also do not explain how much they expect to improve the performance of their system if they improve some of these limitations.
3- Throughout the paper and supplementary information, the authors do not discuss or include the effect of loss in the JTWPA in their analysis or theory. What is the loss of the JTWPA? How does the internal loss in the JTWPA affect the measured squeezing and other metrics? Can you include the effect of loss in the theoretical model?

Minor issues:
1- Can you specify the gain range that the JTWPA achieves for the pump powers in Fig. 4?
2- What is the gain level that is used to produce the results in Fig. 2 and Fig. 3?
3- Can you include further details in section I of the supplementary information in addition to the figure caption? For example, explain the room-temperature equipment. Where are the pump tones applied? What do S, O, L stand for? How are they used for calibration? Why are there two input lines and two output lines?
4- What gain does Fig. 2 in the supplementary material show? The y-axis shows a range of -1.05 to -0.8 dB.
5- Although the JTWPA used in this work is purchased or obtained by a vendor, can you provide a circuit diagram of the JTWPA in the supplementary information so that the readers do not need to go to the citation to find out this important piece of information.
6- What is the value of A and delta omega in Eq. 1 in the supplementary information used in the experiment?

7- In line 672, in the sentence “via more general linear optical transformations”, is optical the correct word here?
8- There is at least one instance of symbol ambiguity or overlap in case you like to resolve it. For example, the Omega symbol in Eq. 21 is also used for the pump frequencies.
9- In line 487, the equation should be 17 and not 18.
10- In line 711, which follows Eq. 48, the theta symbol is defined but it does not appear in that equation.

Reviewer #2

(Remarks to the Author)
In this manuscript, the authors demonstrated the generation of continuous variable cluster states using a JTWPA. Cluster states are a particular class of entangled states which enables universal measurement based quantum computing and have been previously shown in optics as well as microwaves.
the idea of generating cluster states in a Josephson transmission line is interesting for its practicality, since it requires injecting a set of parametric pumps in the TWPA, while alternative ways usually employ more complex protocols with precise pulse timing or complex networks of beam splitters. I have some comments:

1) The authors quantify the quality of their cluster state using Frobenius distance and nullifiers. The minimum nullifier measured is -1dB, while it should be ideally zero for a cluster state. I think it would be beneficial to add more information on error budgeting, how much squeezing can be generated by the TWPA? How much is the impact of HEMT and measurement chain noise and loss?
2) Some recent references on cluster state generation at microwaves are missing, such as:
- Besse, Jean-Claude, et al. "Realizing a deterministic source of multipartite-entangled photonic qubits." Nature communications 11.1 (2020): 4877.
- Sunada, Yoshiki, et al. "Efficient tomography of microwave photonic cluster states." arXiv preprint arXiv:2410.03345 (2024).

3) In the previous references cluster states with fidelity up to 82% are demonstrated. How do the measured Frobenius distance and nullifiers relate to state fidelity? What is the prospect to reach high fidelity with TWPAs, given the loss of such devices, compared to qubit sources for photonic cluster states?

4) TWPAs are typically dispersion engineered to operate as an amplifier. Was the TWPA in the manuscript engineered for multiple pumps or a regular $WM single pump? Or is there no dispersion engineering? How can dispersion be optimized to generate cluster states?

5) Are the authors employing a dissipative generation protocol, similar to reference 32, where dissipation in the TWPA drives the state towards a cluster state? Or is loss detrimental to cluster state generation? Could a dissipative protocol be employed here?

6) The authors extracted the state covariance matrix by subtracting the covariance matrix with the TWPA off from the one with the TWPA on (equation 50 in section IV A). Are there (classical) correlations in the measured covariance matrix with the TWPA in the off state? In general in the case of correlated variables (both from quantum and classical correlations) the noise (co-)variance is not additive. Also a beam splitter model should be use to account for loss in the chain.

Reviewer #3

(Remarks to the Author)
Using a Josephson traveling wave para-amp to generate continuous variable quantum cluster states is a very cool result. Well done. I am confident that the authors understand what they've done pretty well, but I think they could do a better job explaining it to the rest of us-- particularly if they want to publish in Nature Coms, where the target reader may not be extensively familiar with TWPAs or CV quantum computing.

I think the paper could be improved by explaining in better detail the following:
1. What frequencies is the AWG actually sending to the TWPA? Are you sending the mode frequencies \omega_i as well as the pump frequencies \Omega_{i,j}, or just the pump frequencies?
2. Define all of the acronyms in figure 1.
3. Why is this called 3-wave mixing? The linear and star graphs look like 3-wave mixing to me, but the other two look like 4- and 6-wave mixing.
4. What do amplitude and phase quadratures x_l and p_j mean physically? What is actually measured in the lab?
5. Figures two and three have pretty pictures, but I don't know what any of it means. This is related to the previous point. Am I supposed to infer that these are squeezed states? That there is some amount of quantum coherence? What should these plots look like in the ideal case?
6. Why does the color axis on figure 3 extend to -1 if none of the data is negative? Seems like you're just wasting resolution.
7. The text states that "A state is considered a valid cluster state if all nullifier variances are suppressed below the shot noise level (SNL), also known as the quantum limit." Why would this be called a quantum limit? Quantum Limit implies that quantum mechanics prevents the quantity from being any lower. This sounds more like a quantum threshold to me. Like you need to be at least this low to make a legitimate claim of quantum behavior. Plot 4 looks like you're just barely low enough, and only sometimes. Am I interpreting that right?

Version 1:

Reviewer comments:

Reviewer #1

(Remarks to the Author)
The authors have adequately addressed the concerns and comments provided by the referees regarding their manuscript. They have also made significant changes to their manuscript based on these comments, which tremendously improved it. Following these important changes, I recommend the publication of their paper in Nature Communications.

Reviewer #2

(Remarks to the Author)
I think the authors addressed all my comments and provided meaningful comparison metrics to the state of the art. I think the work is suitable for publication.

Reviewer #3

(Remarks to the Author)
This is interesting work because it demonstrates that Continuous Variable Quantum Computation is possible simply by applying carefully selected microwave pump tones to a Josephson traveling wave parametric amplifier. It is quite cleverly done. The methodology is sound. The claims are supported by the work. The authors have addressed all of my concerns with the first draft. I don't see any further flaws in the paper. I think its good to publish.

Open Access This Peer Review File is licensed under a Creative Commons Attribution 4.0 International License, which permits use, sharing, adaptation, distribution and reproduction in any medium or format, as long as you give appropriate credit to the original author(s) and the source, provide a link to the Creative Commons license, and indicate if changes were made.
In cases where reviewers are anonymous, credit should be given to 'Anonymous Referee' and the source.
The images or other third party material in this Peer Review File are included in the article’s Creative Commons license, unless indicated otherwise in a credit line to the material. If material is not included in the article’s Creative Commons license and your intended use is not permitted by statutory regulation or exceeds the permitted use, you will need to obtain permission directly from the copyright holder.
To view a copy of this license, visit https://creativecommons.org/licenses/by/4.0/

We thank the referees for their careful and constructive comments. All points raised have been addressed in the revised manuscript, as detailed below.

For clarity, we explicitly indicate in each response whether changes were made to the manuscript.

The manuscript has been reorganized in accordance with the editorial policies, and revisions have been made to improve clarity and accessibility.

Changes in the revised manuscript are highlighted using the following color code:
Red – text removed
Blue – text moved
Teal – text added
Brown – text replaced

The raw data supporting the findings of this study are available on Figshare under embargo. Referees may access the dataset via the following private link:
[https://figshare.com/s/0c9f014da01c516f342b].

REVIEWER COMMENTS

Reviewer #1 (Remarks to the Author):

In their paper "Programmable Microwave Cluster States via Josephson Metamaterials" the authors experimentally demonstrate the generation of several Continuous Variable (CV) cluster states in the microwave domain. Such states are essential for quantum information processing with continuous variables. They can enable quantum computation that is based on Gaussian states of electromagnetic radiation, deterministic operations, and high-fidelity measurements. To generate these states, the authors use a Josephson metamaterial in the form of a Josephson Traveling wave parametric amplifier (JTWPA). They operate the device in three-wave mixing mode, and apply multiple pump tones to the device simultaneously, which based on their frequencies generate two-mode squeezing states between pairs of nondegenerate modes within the JTWPA bandwidth. By varying the number of applied pump tones and engineering their frequencies, the authors are able to "program" the JTWPA to produce CV cluster states that realize different connectivity configurations between the entangled modes.

In general, the results of the paper are important and interesting to a broad audience. The paper and supplementary information are clear and well written. The measurements are carefully executed and adequately analyzed. I think the paper is a good candidate for publication in Nature Communications after addressing several important issues in a revised version.

Main issues:
1- In their paper, the authors do not provide sufficient perspective or assessment regarding the results they achieve. Although they present several performance metrics such as the nullifier measurement and Frobenius distance, they do not put these values in context for non-expert readers to understand whether they are good or not. For example, can you compare these values to the ones measured in the optical domain? Are these values sufficient for performing scalable or even basic CV quantum computation in the microwave domain? How far off are they?

We thank the referee for the comment. This point has been addressed by expanding the discussion in **Sec. DISCUSSION**.

A dedicated subsection entitled **"Comparison with the state of the art"** has been added to provide quantitative benchmarking against optical continuous-variable platforms.

From the main text:

"In our experiment, we observed nullifier squeezing of 0.8 dB, 0.4 dB, 0.4 dB, and 0.2 dB for the linear, cyclic, star, and fully connected clusters, respectively, with a reduction in squeezing for topologies requiring more pump tones, consistent with previous results in the microwave regime [12,32]. These values fall within the range reported in optical continuous-variable studies for clusters of comparable size: bulk OPO-based implementations reach up to ~ 3 dB of squeezing for linear, cyclic, and star configurations [22], while integrated microcomb chips achieve around ~ 2 dB [8]. Although our microwave platform exhibits lower squeezing, its in-situ programmability and compatibility with superconducting hardware provide a complementary approach toward scalable measurement-based continuous-variable quantum computation."

The implications for scalability and computational applicability are further discussed in the subsection **"Future perspectives"**, where we explicitly address whether the achieved squeezing levels are sufficient for basic or scalable CV quantum computation in the microwave domain.

From the main text:
"The achieved nullifier squeezing is sufficient to implement elementary continuous-variable protocols such as entanglement swapping and quantum teleportation in the microwave domain [33] provided that appropriate Bell-type measurements are performed. By contrast, universal and fault-tolerant CV quantum computation requires substantially larger cluster states (with a number of modes that scales with the circuit width and depth) and higher squeezing (on the order of 15 dB), together with a non-Gaussian resource. These requirements highlight that significant advances in cluster size and achievable squeezing will be necessary to reach this regime in the microwave domain [29,34–36]"

2- In their paper, the authors cite several limitations of their device and setup and mention several ways to overcome them but do not provide any quantitative estimates of these limitations or explain how they are affecting the measured performance and to what extent. They also do not explain how much they expect to improve the performance of their system if they improve some of these limitations.

We thank the referee for the comment. This point has been addressed in **Sec. DISCUSSION**, subsection **"Future perspectives"**, where we provide a detailed analysis of the current limitations and outline possible routes toward improvement.

From the main text:
"Current limitations arise from both the device under test (DUT) and the measurement chain. At the device level, they include limited squeezing generation, potentially worsened by residual higher order harmonics products that contribute to losses in the JTWPA [32]. A potential improvement is the use of a left-handed JTWPA, which is expected to suppress unwanted mixing processes and achieve higher squeezing levels [38,39]. Experimental limitations arise from the use of a 3 K HEMT as the first amplification stage and from room-temperature pump-tone filtering. These issues could be mitigated by implementing a quantum-limited amplifier as the first stage and by introducing cryogenic pump filtering to improve pump isolation and measurement stability. A 3 K HEMT typically adds on the order of 20 noise quanta, whereas quantum-limited amplifiers add a minimum of 1/2 quantum, which would reduce the required acquisition time and mitigate the impact of system drifts [40]."

At present, fully quantitative projections of achievable improvements cannot be included in the manuscript, as the relevant experimental and theoretical results are not yet published. However, indicative benchmarks have been reported in recent conference presentations and private communications.

Regarding the use of a left-handed JTWPA, preliminary results presented at the workshop *"Microwave Amplifiers in the Quantum Regime"* (Les Houches) by Archana Kamal (Northwestern University) indicate that up to ~10 dB two-mode squeezing could be achieved, substantially higher than the ~3 dB obtained with right-handed TWPAs. These results are currently being prepared for publication and are cited as a private communication (A. Kamal, private communication). Similarly, experimental squeezing of approximately 5 dB has been reported in an ongoing doctoral thesis (Cappelli, PhD thesis, in preparation) and is cited [39] as a private communication pending publication.
Regarding the use of a JTWPA as the first-stage amplifier and the introduction of cryogenic pump filtering, preliminary results have been presented at the *"Global Physics Summit"* (Anaheim) and at

the workshop *"Microwave Amplifiers in the Quantum Regime"* (Les Houches) by Logan Howe (NIST). These results are currently being prepared for publication and are cited as a private communication (A. Giachero, private communication).

3- Throughout the paper and supplementary information, the authors do not discuss or include the effect of loss in the JTWPA in their analysis or theory. What is the loss of the JTWPA? How does the internal loss in the JTWPA affect the measured squeezing and other metrics? Can you include the effect of loss in the theoretical model?

We thank the referee for the comment. The effect of loss has now been incorporated into the theoretical model, as described in **Sec. 3.6** of the revised Supplementary Material. This section also discusses the impact of losses on the measured squeezing and other relevant metrics.

The experimentally measured losses of the JTWPA have been reported in **Sec. 4.8** of the Supplementary Material.

From the Supplementary Material:
"The theoretical covariance matrices were computed using the formalism presented in Section 3.4, including the effects of JTWPA losses as described in Section 3.6. The measured total loss of the device and its packaging, including connections to electromechanical switches, is $\eta = -1$ dB".

Minor issues:
1- Can you specify the gain range that the JTWPA achieves for the pump powers in Fig. 4?

We thank the referee for the comment. The gain range corresponding to the pump powers used in **Fig. 4**, averaged over the four nodes' frequencies, spans approximately 0.1–2.6 dB, as shown in the newly added **Fig. 5** of the Supplementary Material. Further details on the gain estimation procedure can be found in **Sec. 4.4** of the Supplementary Material.

2- What is the gain level that is used to produce the results in Fig. 2 and Fig. 3?

We thank the referee for the comment. The results shown in **Figs. 2 (now Fig. 4) and 3** were obtained at a pump power of –24 dBm, corresponding to an average JTWPA gain of approximately 0.7, 1.0, 0.8, and 1.4 dB for the linear, cyclic, star, and fully connected topologies, respectively (see the newly added **Fig. 5** of the Supplementary Material). Further details on the gain estimation procedure can be found in **Sec. 4.4** of the Supplementary Material.

3- Can you include further details in section I of the supplementary information in addition to the figure caption? For example, explain the room-temperature equipment. Where are the pump tones applied? What do S, O, L stand for? How are they used for calibration? Why are there two input lines and two output lines?

We thank the referee for the comment. Further description of the setup shown in **Fig. 1** of the Supplementary Material has been added as a figure description, here reported:

"The experiment is hosted in a dilution refrigerator, where two input lines are coupled to two output lines at the mixing chamber plate. Two cryogenic electromechanical switches allow for Short-Open-Load-Reciprocal (SOLR) deembedding and in situ noise calibration using a variable temperature stage (VTS) connected to a thermalized 20 dB attenuator [1,2].
At room temperature, two channels of an Arbitrary Waveform Generator (AWG) are used for pump generation, while a channel of a downconverter (DNC) and a digitizer (DIG) is used for heterodyne detection. The AWG, DNC, and DIG are components of a digital quantum control system (Keysight Technologies, Santa Rosa, CA, USA).
Two additional switches allow routing to a vector network analyzer (VNA) for preliminary characterization."

Regarding the referee's question about the two input and two output lines, these are used when characterizing a two-port device in both directions, allowing the full reconstruction of all four S-parameters using the SOLR calibration method.

4- What gain does Fig. 2 in the supplementary material show? The y-axis shows a range of -1.05 to -0.8 dB.

We thank the referee for the comment. In the previous version, the plotted quantity in **Fig.2 (now Fig. 3)** of Supplementary Material was erroneously referred to as "gain." In reality, it corresponds to the transmission coefficient **S21** of the Device Under Test (DUT). The y-axis values (−1.05 to −0.8 dB) are negative because they represent the transmission from input to output, including uncompensated cables, electromechanical switches, and the device package, which introduces additional losses.

To better guide the reader in interpreting this image, the y-axis of **Fig. 3** has been relabeled as **S21**, and a more detailed figure description has been inserted, here reported:
"Transmission coefficient S21 of the device under test (DUT) as a function of signal frequency, measured for six pump frequencies Ω(k) at fixed pump power PΩ = −21 dBm.
The DUT consists of the JTWPA integrated in its package and connected to electromechanical switches through uncompensated cables. The S-parameters are referenced to the electromechanical switches and are obtained via a Short–Open–Load–Reciprocal (SOLR) calibration procedure. The amplifier is flux-biased at Φ = 0.378 Φ0 to operate predominantly in the three-wave mixing regime. Dashed vertical lines mark the resonant modes of the investigated cluster. The inset displays the phase of the transmission coefficient S21."

5- Although the JTWPA used in this work is purchased or obtained by a vendor, can you provide a circuit diagram of the JTWPA in the supplementary information so that the readers do not need to go to the citation to find out this important piece of information.

We thank the referee for the comment. A detailed description of the JTWPA circuit and its schematic has been added in **Sec. 2.1** of the Supplementary Material.

6- What is the value of A and delta omega in Eq. 1 in the supplementary information used in the experiment?

We thank the referee for the comment. In the experiment, the parameters used in **Eq. (1)** of the Supplementary Information were chosen as follows:
$\Delta\omega = 2\pi \cdot 1.25$ MHz, $A = 2\pi$ MHz.
These values have been added explicitly to **Sec. 2.4** of the Supplementary Material for clarity.

7- In line 672, in the sentence "via more general linear optical transformations", is optical the correct word here?

We thank the referee for the comment. The phrase "via more general linear optical transformations" has been replaced with "linear transformations" to more accurately reflect the generality of the operations, which are not limited to optical implementations.

8- There is at least one instance of symbol ambiguity or overlap in case you like to resolve it. For example, the Omega symbol in Eq. 21 is also used for the pump frequencies.

We thank the referee for the comment. This issue has been resolved by renaming Ω in **Eq. (21)** to J, thereby eliminating any overlap with the pump frequencies.

9- In line 487, the equation should be 17 and not 18.

We thank the referee for the comment. The reference has been corrected.

10- In line 711, which follows Eq. 48, the theta symbol is defined but it does not appear in that equation.

We thank the referee for the comment. The definition of θ in line 711 has been removed.

Reviewer #2 (Remarks to the Author):

In this manuscript, the authors demonstrated the generation of continuous variable cluster states using a JTWPA. Cluster states are a particular class of entangled states which enables universal measurement based quantum computing and have been previously shown in optics as well as microwaves.
the idea of generating cluster states in a Josephson transmission line is interesting for its practicality, since it requires injecting a set of parametric pumps in the TWPA, while alternative ways usually employ more complex protocols with precise pulse timing or complex networks of beam splitters. I have some comments:

1) The authors quantify the quality of their cluster state using Frobenius distance and nullifiers. The minimum nullifier measured is -1dB, while it should be ideally zero for a cluster state. I think it would be beneficial to add more information on error budgeting, how much squeezing can be generated by the TWPA? How much is the impact of HEMT and measurement chain noise and loss?

We thank the referee for the comment. We first clarify that the quantity reaching −1 dB corresponds to the nullifier variance normalized to the vacuum variance. In the ideal cluster-state limit, the nullifier operator vanishes, while its variance—expressed in dB relative to vacuum—tends to −∞, not to zero. Therefore, the measured −1 dB reflects finite squeezing below vacuum, and does not imply proximity to zero absolute variance.

The two-mode squeezing characterization of the JTWPA as a function of single pump power has been added in **Sec. 2.3** of the Supplementary Material, showing up to 3 dB of two-mode squeezing below vacuum.
An uncertainty budget study has been added in **Sec. 4.3** of the Supplementary Material. **Fig. 2** of the main paper has been updated to include the estimated standard deviations on the plotted data. Regarding the measurement chain, the impact of the 3 K HEMT amplifier and other elements has been discussed in a new paragraph of **Sec. DISCUSSION**, subsection **Future perspectives**.

From the text:
"Experimental limitations arise from the use of a 3 K HEMT as the first amplification stage and from room-temperature pump-tone filtering. These issues could be mitigated by implementing a quantum-limited amplifier as the first stage and by introducing cryogenic pump filtering to improve pump isolation and measurement stability. A 3 K HEMT typically adds on the order of 20 noise quanta, whereas quantum-limited amplifiers add a minimum of 1/2 quantum, which would reduce the required acquisition time and mitigate the impact of system drifts [40]."

2) Some recent references on cluster state generation at microwaves are missing, such as:
- Besse, Jean-Claude, et al. "Realizing a deterministic source of multipartite-entangled photonic qubits." Nature communications 11.1 (2020): 4877.
- Sunada, Yoshiki, et al. "Efficient tomography of microwave photonic cluster states." arXiv preprint arXiv:2410.03345 (2024).

We thank the referee for the comment. The references suggested by the referee (Besse et al., 2020; Sunada et al., 2024) have been added to the **Introduction** as examples of cluster states generated in discrete-variable (qubit) microwave systems.

From the text:
"However, transitioning cluster state generation to the microwave regime holds great promise, particularly in the context of superconducting circuits, both in discrete and CV architectures [10–12]"

3) In the previous references cluster states with fidelity up to 82% are demonstrated. How do the measured Frobenius distance and nullifiers relate to state fidelity? What is the prospect to reach high fidelity with TWPAs, given the loss of such devices, compared to qubit sources for photonic cluster states?

We thank the referee for the comment. For this point, the only change made in the manuscript is the renaming of the Frobenius distance to Frobenius Similarity (denoted S_F) to avoid possible confusion with the fidelity. The following explanation is provided to clarify the role of the different figures of merit in response to the referee.

The reviewer raises an important benchmarking issue. Discrete-variable (DV) cluster states, such as those cited, consist of entangled qubits and are naturally characterized by fidelity or stabilizer expectation values. Continuous-variable (CV) cluster states instead are multimode Gaussian states of bosonic modes, where the relevant resource for MBQC is the suppression of nullifier variances, directly quantifying how well the cluster graph constraints are satisfied [29,5,25,7].
All these figures of merit derive from the covariance matrix: nullifiers probe the graph-defining correlations, Gaussian fidelity measures global overlap with a target state, and the Frobenius Similarity provides a numerically robust, model-independent quantification of agreement between experimental and theoretical covariance matrices. We therefore use nullifiers to certify the operational resource and the Frobenius Similarity to validate the overall correlation structure.
Regarding scalability and achievable fidelity, TWPAs introduce distributed loss and added noise, which limit attainable squeezing and thus Gaussian fidelity. However, they operate deterministically and over large bandwidths, enabling the generation of thousands of modes in a single spatial channel. In contrast, photonic qubit cluster states rely on probabilistic entangling operations, and losses, imperfect gates, and synchronization errors accumulate rapidly with system size. For one-way quantum computing, high fidelity alone is insufficient; large-scale entanglement is also required. In this perspective, TWPAs offer a promising route toward deterministic, large-scale CV cluster states with operationally useful squeezing levels.

4) TWPAs are typically dispersion-engineered to operate as an amplifier. Was the TWPA in the manuscript engineered for multiple pumps or a regular $WM single pump? Or is there no dispersion engineering? How can dispersion be optimized to generate cluster states?

We thank the referee for the comment. A detailed description of the JTWPA has been added in **Sec. 2.1** of the Supplementary Material.
The JTWPA used is a standard 3WM single-pump amplifier.

From the text:
"The device investigated in this study is a pre-commercial AI-TWPA-C [4] from Arctic Instruments, featuring a central conductor composed of a series array of flux-tunable SNAILs(Superconducting Nonlinear Asymmetric Inductive eLements) (see Fig. 2) [5]. A uniformDC flux applied through an on-chip bias line maximizes three-wave mixing (3WM), while leaving a residual four-wave mixing (4WM) component and, consequently, a Kerr effect.
Periodically integrated spiral resonators and capacitors are used to engineer the line dispersion, producing a superlinear response in the frequency range of the second pump harmonic under typical operating conditions. This makes its direct generation inefficient, yet sufficient for back-conversion to the pump, which imparts a controlled phase shift (effective Kerr effect) essential for maintaining broadband phase matching and gain. The AI-TWPA-C is fabricated using multilayer niobium Josephson technology, with Nb/Al–AlOx/Nb trilayers forming the junctions. The primary niobium layer constructs the CPW ground planes and capacitors, while a secondary niobium layer carries the meandering flux-bias line and the top electrodes of the shunt capacitors. Thin normal-metal films define resistive terminations to ground, stabilizing the flux bias."

About the reviewer's question on squeezing optimization through dispersion engineering: no modifications to the manuscript were necessary for this point. In general, optimizing dispersion for cluster-state generation involves ensuring that only the desired parametric processes are phase-matched while minimizing higher-order nonlinear mixing, thereby enabling controlled entanglement between specific sets of modes.

5) Are the authors employing a dissipative generation protocol, similar to reference 32, where dissipation in the TWPA drives the state towards a cluster state? Or is loss detrimental to cluster state generation? Could a dissipative protocol be employed here?

We thank the referee for the comment. In our work, loss is detrimental for squeezing generation. This point has been discussed in the newly added **Sec. 3.6** of the Supplementary Material.

For clarity in response to the referee, Ref. [37] employs a dissipative generation protocol using a coupled resonator to drive the system toward the cluster state, while the JTWPA in our experiment does not act as a dissipative source. Implementing a dissipative stabilization protocol would therefore require a different architecture and is not part of the present scheme.

6) The authors extracted the state covariance matrix by subtracting the covariance matrix with the TWPA off from the one with the TWPA on (equation 50 in section IV A). Are there (classical) correlations in the measured covariance matrix with the TWPA in the off state? In general in the case of correlated variables (both from quantum and classical correlations) the noise (co-)variance is not additive. Also a beam splitter model should be use to account for loss in the chain.

We thank the referee for the comment. **Sec. 4.1** of the Supplementary Material has been complemented with additional information to address the reviewer's question.
The amplification chain is modeled to include signal gain and added noise, while JTWPA losses are treated with an effective beam-splitter model.

From the text:
"In the notation defined in Eq. [20], the quadrature operators after the amplification chain for the j-th mode, I_j and Q_j, can be modeled as:
$I_j + i Q_j = \sqrt{2} \times [\sqrt{G_{SYS,j}} \times a_j + \sqrt{G_{SYS,j} - 1} \times h_j] \approx \sqrt{2 \times G_{SYS,j}} \times (a_j + h_j)$, for $G_{SYS,j} \gg 1$.
Here, G_SYS,j denotes the system gain for the j-th spectral mode, a_j describes the j-th mode at the JTWPA output, and h_j is an effective bosonic operator describing the noise added by the amplification chain."

Regarding the question about classical correlations and **Eq.(50) (now Eq.(58))**:

From the text:
"The reference-state subtraction is a standard method to cancel out the noise added by the amplification chain, assuming the mode described by the operator ˆhj is in a thermal state [9,10]. This assumption is confirmed by the off-diagonal elements of ΓexpOFF, all of which have Pearson coefficients below 0.005, consistent with a diagonal thermal covariance matrix."

Reviewer #3 (Remarks to the Author):

Using a Josephson traveling wave para-amp to generate continuous variable quantum cluster states is a very cool result. Well done. I am confident that the authors understand what they've done pretty well, but I think they could do a better job explaining it to the rest of us-- particularly if they want to publish in Nature Coms, where the target reader may not be extensively familiar with TWPAs or CV quantum computing.

I think the paper could be improved by explaining in better detail the following:
1. What frequencies is the AWG actually sending to the TWPA? Are you sending the mode frequencies \omega_i as well as the pump frequencies \Omega_{i,j}, or just the pump frequencies?

We thank the referee for the comment. The AWG sends only the pump tones at frequencies Ω_{jl}, which drive the JTWPA in the three-wave mixing regime.
This point has been clarified in the updated description of **Fig. 1**, which now explicitly explains the frequency-domain protocol and the corresponding pump configurations for generating cluster-state edges.

From the text:
"Programmable generation of microwave cluster states using a Josephson Traveling-Wave Parametric Amplifier (JTWPA). (a) Frequency-domain continuous-variable (CV) cluster-state protocol. Multiple non-equidistant pump tones at frequencies Ω_{jl} drive the JTWPA in the three-wave mixing regime, implementing two-mode squeezing interactions between vacuum modes at frequencies ω_j and ω_l. The interaction transforms input vacuum fluctuations into two-mode squeezed vacuum states, while thermal noise from room-temperature (RT) components is suppressed by cryogenic attenuation. The detection scheme employs a pump ON/OFF reference protocol to subtract the added noise of the readout chain. (b) Block diagram of the experimental setup, including an arbitrary waveform generator (AWG) for pump synthesis, cryogenic attenuators (ATT) to reduce RT thermal noise, the JTWPA, an isolator to prevent back-action, a cryogenic HEMT amplifier followed by a LNA, a low-pass filter (LPF) to suppress pump leakage, and a multiplexed heterodyne detection stage. Inset: example cluster topologies and corresponding pump configurations. Each edge between modes (j, l) is implemented by applying a pump tone at frequency $\Omega_{jl} = \omega_j + \omega_l$."

2. Define all of the acronyms in figure 1.

We thank the referee for the comment. All acronyms in **Fig. 1** (AWG, ATT, JTWPA, HEMT, LNA, LPF) have been defined in the updated figure caption.

From the text:
"Programmable generation of microwave cluster states using a Josephson Traveling-Wave Parametric Amplifier (JTWPA). (a) Frequency-domain continuous-variable (CV) cluster-state protocol. Multiple non-equidistant pump tones at frequencies Ω_{jl} drive the JTWPA in the three-wave mixing regime, implementing two-mode squeezing interactions between vacuum modes at frequencies ω_j and ω_l. The interaction transforms input vacuum fluctuations into two-mode squeezed vacuum states, while thermal noise from room-temperature (RT) components is suppressed by cryogenic attenuation. The detection scheme employs a pump ON/OFF reference protocol to subtract the added noise of the readout chain. (b) Block diagram of the experimental setup, including an arbitrary waveform generator (AWG) for pump synthesis, cryogenic attenuators (ATT) to reduce RT thermal noise, the JTWPA, an isolator to prevent back-action, a cryogenic HEMT amplifier followed by a LNA, a low-pass filter (LPF) to suppress pump leakage, and a multiplexed heterodyne detection stage. Inset: example cluster topologies and corresponding pump configurations. Each edge between modes (j, l) is implemented by applying a pump tone at frequency $\Omega_{jl} = \omega_j + \omega_l$."

3. Why is this called 3-wave mixing? The linear and star graphs look like 3-wave mixing to me, but the other two look like 4- and 6-wave mixing.

We thank the referee for the comment. The term "three-wave mixing" (3WM) refers to the nonlinear interaction implemented by the JTWPA, where a single pump photon at frequency Ω_{jl} mediates the creation of two photons at mode frequencies ω_j and ω_l, satisfying $\Omega_{jl}=\omega_j+\omega_l$. This point is now clarified in the description of **Fig. 1**.

From the text:
"Programmable generation of microwave cluster states using a Josephson TravelingWave Parametric Amplifier (JTWPA). (a) Frequency-domain continuous-variable (CV) cluster-state protocol. Multiple non-equidistant pump tones at frequencies Ω_{jl} drive the JTWPA in the three-wave mixing regime, implementing two-mode squeezing interactions between vacuum modes at frequencies ω_j and ω_l . The interaction transforms input vacuum fluctuations into two-mode squeezed vacuum states, while thermal noise from room-temperature (RT) components is suppressed by cryogenic attenuation. The

detection scheme employs a pump ON/OFF reference protocol to subtract the added noise of the readout chain. (b) Block diagram of the experimental setup, including an arbitrary waveform generator (AWG) for pump synthesis, cryogenic attenuators (ATT) to reduce RT thermal noise, the JTWPA, an isolator to prevent back-action, a cryogenic HEMT amplifier followed by a LNA, a low-pass filter (LPF) to suppress pump leakage, and a multiplexed heterodyne detection stage. Inset: example cluster topologies and corresponding pump configurations. Each edge between modes (j,l) is implemented by applying a pump tone at frequency $\Omega jl = \omega j + \omega l$."

4. What do amplitude and phase quadratures x_l and p_j mean physically? What is actually measured in the lab?

We thank the referee for the comment. This point has been addressed in more detail in **Sec. RESULTS, Cluster state verification**.

From the text:
"To experimentally verify this condition, we perform heterodyne detection on each frequency mode ω_j. The measurement yields the in-phase (I) and quadrature (Q) voltage components of the microwave field, from which we construct the covariance matrix Γ at the JTWPA output, defined on the quadrature vector $R = (x_1, p_1, \ldots, x_N, p_N)^T$. Building on this, we compute the nullifier variances using Equation 2."

Further details are provided in **Sec. METHODS**:

From the text:
"We perform heterodyne detection on each frequency mode, properly tuning a set of Local Oscillators (LO) and exploiting a digital down-conversion scheme to extract correlations between different quadratures over time windows synchronized with the pump tone cycle. Each acquisition consists of two consecutive stages: one with the pump tones activated (ON) and one with the pumps turned off (OFF), under otherwise identical experimental conditions (see Figure 4(a)). By repeating this procedure 5×10^6 times, we reconstruct two covariance matrices, Γ_exp^ON and Γ_exp^OFF, computed from the scaled quadrature vector $M = (i_1, q_1, \ldots, i\_N, q\_N)^T$, where i_j and q_j are obtained by calibrating the raw in-phase and quadrature voltage signals I_j and Q_j from heterodyne detection into the photon basis. The covariance matrix at the JTWPA output is then obtained via a reference state subtraction method (see Figure 4(b,c)): Γ_θ = Γ_exp^ON − Γ_exp^OFF + σ_in (3). Here, σ_in is the covariance matrix of the thermal state at cryogenic temperature. From the reconstructed covariance matrix Γ_θ, we recover the cluster-state covariance matrix Γ in the quadrature basis $R = (x_1, p_1, \ldots, x\_N, p\_N)^T$ via numerical optimization of local transformations (see Figure 3(b–f)), maximizing the off-diagonal x–p correlations."

5. Figures two and three have pretty pictures, but I don't know what any of it means. This is related to the previous point. Am I supposed to infer that these are squeezed states? That there is some amount of quantum coherence? What should these plots look like in the ideal case?

We thank the referee for the comment. **Fig. 2** (now **Fig. 4**) describes the method used to reconstruct the state covariance matrix.
Fig. 3 shows the reconstructed covariance matrices and emphasizes, in the second row, the correlation elements.

A more detailed description of **Fig. 4** and **Fig. 3** is provided in **Sec. METHODS, Covariance matrix reconstruction**.

From the text:
"We perform heterodyne detection on each frequency mode, properly tuning a set of Local Oscillators (LO) and exploiting a digital down-conversion scheme to extract correlations between different quadratures over time windows synchronized with the pump tone cycle. Each acquisition consists of two consecutive stages: one with the pump tones activated (ON) and one with the pumps turned off

(OFF), under otherwise identical experimental conditions (see Figure 4(a)). By repeating this procedure 5×10^6 times, we reconstruct two covariance matrices, Γ_{exp}^{ON} and Γ_{exp}^{OFF}, computed from the scaled quadrature vector $M = (i_1, q_1, \ldots, i_N, q_N)^T$, where i_j and q_j are obtained by calibrating the raw in-phase and quadrature voltage signals I_j and Q_j from heterodyne detection into the photon basis. The covariance matrix at the JTWPA output is then obtained via a reference state subtraction method (see Figure 4(b,c)): $\Gamma_\theta = \Gamma_{exp}^{ON} - \Gamma_{exp}^{OFF} + \sigma_{in}$ (3). Here, σ_{in} is the covariance matrix of the thermal state at cryogenic temperature. From the reconstructed covariance matrix Γ_θ, we recover the cluster-state covariance matrix Γ in the unrotated quadrature basis $R = (x_1, p_1, \ldots, x_N, p_N)^T$ via numerical optimization of local phase rotations (see Figure 3(b–f)), maximizing the off-diagonal $p - x$ correlations."

To further clarify the sequence of measured and reconstructed quadratures ($I,Q \rightarrow i,q \rightarrow x,p$), the scaled quadratures displayed in **Fig. 4** have been relabeled as i and q.

The **DISCUSSION, Programmability** section has been enriched with the following text describing **Fig. 3**:

From the text:
"The programmability of the cluster states is further confirmed by the corresponding network graphs, which match the programmed topology (see Figure 3). The reconstructed graphs covariance matrices describing the cluster states are quantitatively compared with theoretical predictions using the normalized Frobenius similarity (SF) (see Supplementary Information), with the worst-case deviation below 2%, demonstrating excellent agreement with the target graph".

The ideal counterpart of the covariance matrices is illustrated in **Fig. 10** of the Supplementary Material.

6. Why does the color axis on figure 3 extend to -1 if none of the data is negative? Seems like you're just wasting resolution.

We thank the referee for the comment. No modifications to the manuscript were necessary for this point.

The color axis in **Fig. 3** extends to −1 to remain consistent with the raw, non-optimized covariance matrices, which can include negative elements (see **Fig. 4**). This scale choice emphasizes that after optimization the covariance matrices are nearly entirely positive, highlighting the effectiveness of the nullifier minimization and the cluster-state reconstruction.

7. The text states that "A state is considered a valid cluster state if all nullifier variances are suppressed below the shot noise level (SNL), also known as the quantum limit." Why would this be called a quantum limit? Quantum Limit implies that quantum mechanics prevents the quantity from being any lower. This sounds more like a quantum threshold to me. Like you need to be at least this low to make a legitimate claim of quantum behavior. Plot 4 looks like you're just barely low enough, and only sometimes. Am I interpreting that right?

We thank the referee for the comment.
The reference to the "Quantum Limit" has been removed from **Fig. 4 (now Fig. 2)** and from the text in the **Results** section, subsection "**Cluster state verification**." This threshold is now referred to simply as the Shot Noise Level (SNL).
The referee is correct that the measured nullifier variances in **Fig. 4 (now Fig. 2)** are close to the SNL, reflecting the finite squeezing achieved in the experiment. A more detailed discussion of these results—including the interpretation of values near the SNL, strategies for increasing squeezing, and comparison with the state of the art—has been added to the **Discussion** section.
